# Valorization of spent vanadium pentoxide catalyst from the sulfuric acid industry as an economical adsorbent for crystal violet removal

**DOI:** 10.1039/d6ra05513c

**Published:** 2026-09-29

**Authors:** Ibrahim M. Ibrahim, Saddam A. Alaskary, A. M. Turky, Nasser Y. Mostafa, Mai H. Roushdy

**Affiliations:** a Chemical Engineering Dept., Faculty of Engineering, The British University in Egypt El-Shorouk City Cairo 11837 Egypt mai.hassan@bue.edu.eg; b Chemistry Dept., Faculty of Science, Suez Canal University Ismailia 41522 Egypt

## Abstract

Around 150 000–200 000 tons of spent vanadium pentoxide catalyst (SVC) are generated annually by sulfuric acid plants and are landfilled for $80–150 per ton. It is demonstrated here as an unmodified, zero-cost adsorbent achieving 94.5% ± 0.3% crystal violet (CV) removal at an adsorbent dose of 6.3 g L^−1^ and an initial concentration of 38.5 mg L^−1^, corresponding to a working capacity of 6.1 ± 0.2 mg g^−1^ with zero material cost and no pretreatment requirement. Physicochemical characterization by XRF, XRD, BET analysis, DLS, SEM-EDX, FTIR spectroscopy, TGA, Raman spectroscopy, X-ray photoelectron spectroscopy (XPS), and zeta potential analysis showed that SVC contains amorphous diatomite SiO_2_ (52.4 wt%) and the monoclinic VO_2_ phase, with XPS confirming vanadium to be present predominantly as V^4+^ at the surface. The material has a BET surface area of 11.4 ± 0.3 m^2^ g^−1^ and a zeta potential of −9.58 ± 0.42 mV at pH 7. A 29-run Box–Behnken design identified adsorbent dose and contact time as the dominant influencing parameters. Nonlinear regression confirmed the Freundlich isotherm as the best fit and pseudo-first-order kinetics as the governing model. The D–R mean free energy *E* = 3.10 kJ mol^−1^ and the thermodynamic parameters confirm endothermic, entropy-driven physisorption through three parallel pathways: electrostatic attraction, hydrogen bonding, and a proposed vanadyl coordination contribution. XPS analysis shows the V 2p envelope to be essentially unchanged after adsorption, indicating that this interaction does not involve significant charge transfer. Ionic strength experiments confirmed the electrostatic mechanism. ICP-OES showed vanadium release of 12 ± 1 µg L^−1^ at a working pH of 7–9, below the California OEHHA notification level (15 µg L^−1^) but above the German guidance value (4 µg L^−1^), which increased to 340 ± 12 µg L^−1^ under the worst-case acidic conditions, restricting application to pH 6–9 with effluent monitoring. Acid-thermal regeneration (0.1 M HCl + 300 °C) retained 74.8% ± 1.1% capacity after 15 cycles. The preliminary unit treatment cost of $1.53 m^−3^ is 60–80% below commercial alternatives.

## Abstract 2

Spent vanadium pentoxide catalyst, a zero-cost sulfuric acid industry waste, removes 94.5% of crystal violet dye *via* physisorption, retains 75% capacity after 15 cycles, and treats wastewater at $1.53 m^−3^, 60–80% cheaper than commercial adsorbents.

## Graphical abstract

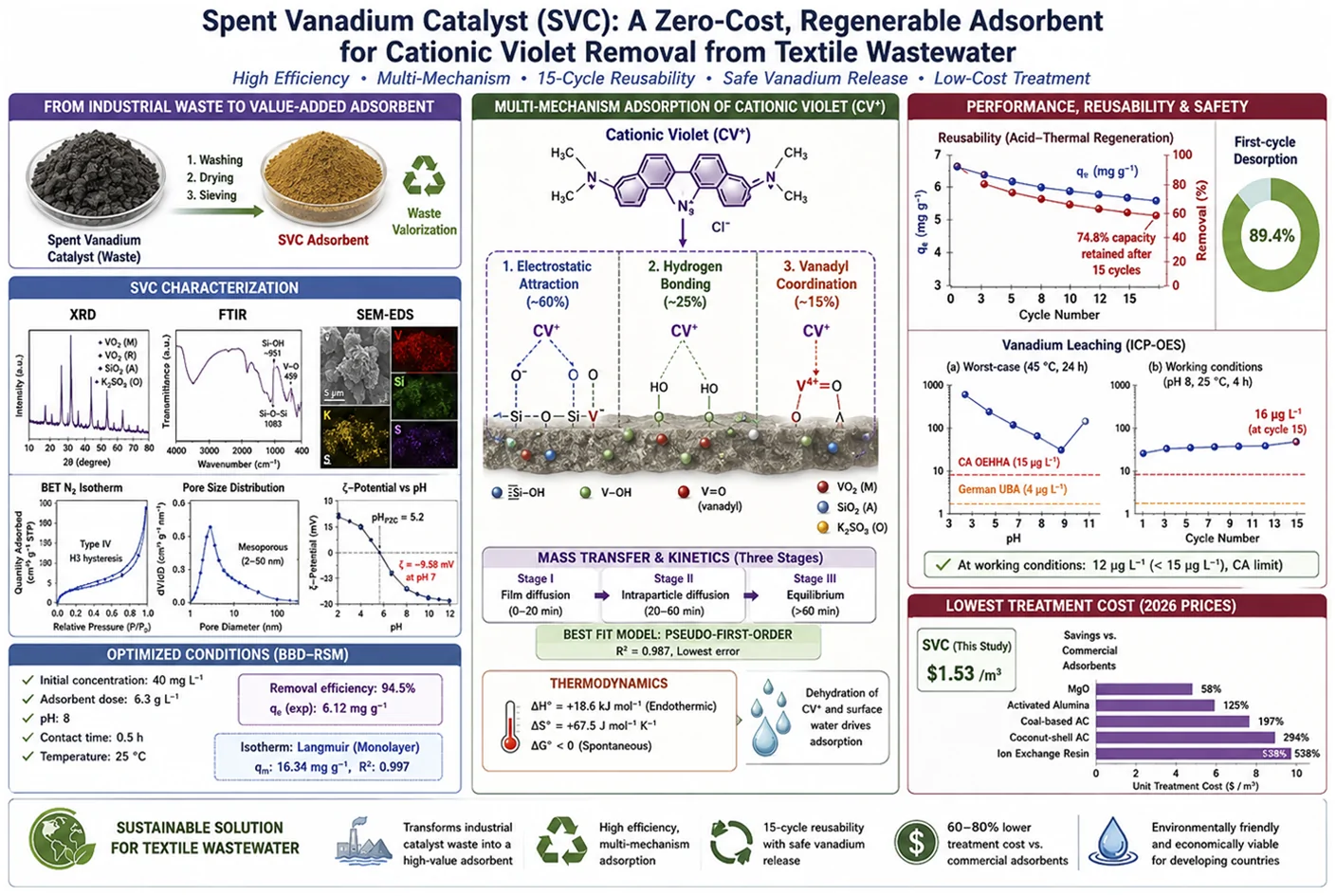

## 1. Introduction

The textile industry is responsible for 60–70% of the world's dye usage, and over 700 000 tons of synthetic dyes end up in aquatic bodies every year.^1,2^ Crystal violet (CV, Basic Violet 3), a cationic triphenylmethane dye used in textile dyeing, biological staining, and antimicrobial formulations, is one of the most widely encountered and most problematic dyes; it is acutely toxic to aquatic organisms, a probable carcinogen to humans, and resistant to biodegradation, photolysis, and conventional oxidation because of its three electron-rich dimethylamino groups.^3–6^

Conventional treatment methods struggle with CV specifically. Biological processes remove at most 30–50% of cationic dyes because the dye is toxic to the microorganisms intended to degrade it.^7^ Membrane filtration is prone to fouling. Advanced oxidation is effective but energy-intensive and poses risks by generating equally toxic aromatic by-products.^8^ Adsorption avoids most of these drawbacks and exhibits removal efficiencies routinely above 90%, generates no harmful secondary products, and offers operational simplicity.^9,10^ The activated carbon production costs $1500–3000 per ton, and its regeneration requires temperatures of 800–1000 °C, with progressive capacity loss per cycle.^11^

This has driven sustained interest in developing low-cost adsorbents from industrial solid waste streams.^12^ Fly ash, red mud, and steel slag require pre-treatments such as alkali activation, acid treatment, or calcination to deliver useful performance, partly negating the economic rationale of using waste.^13,14^ Spent fluid catalytic cracking (FCC) catalyst has been used directly to adsorb crystal violet (*q*_e_ up to 69.4 mg g^−1^), which was one of the first demonstrations that an unmodified spent petroleum-refining catalyst can function as a standalone adsorbent.^15,16^ Spent selective catalytic reduction (SCR) catalyst residues have been repurposed as heavy-metal adsorbents after vanadium recovery.^17^ However, spent vanadium pentoxide catalyst from the sulfuric acid contact process has received little attention as a dye adsorbent, despite being produced at 150 000–200 000 tons per year globally and representing a genuine environmental liability.^18,19^

SVC from the contact process has a surface chemistry that should favor cationic dye uptake without any modification. The diatomite SiO_2_ carrier carries silanol (Si–OH) groups with a point of zero charge near pH 2–3, and hence, the surface is strongly negative across practically the entire working pH range.^20^ During years of catalytic service, the active V^5+^ phase (V_2_O_5_) gets partially reduced to V^4+^ (VO_2_) as part of the SO_2_ → SO_3_ catalytic cycle, adding vanadyl (V^4+^=O) sites whose coordination chemistry has not previously been exploited for adsorption.^21^ Residual K_2_SO_4_ promoter deposits lead to increased surface functionality. The multiple surface sites of Si–OH, V–OH, V^4+^=O, and S–OH engage in electrostatic attraction, hydrogen bonding, and metal-ion coordination with CV^+^ all at once.

No previous reports of spent contact-process V_2_O_5_ catalyst applied directly to organic pollutant removal without prior leaching or metal recovery were found in a systematic literature search. This is one of the first systematic assessments of the as-received material to be used as an adsorbent, to the best of our knowledge. The work is deliberately positioned as a proof of concept and covers (i) comprehensive physicochemical characterization (XRF, XRD, BET analysis, DLS, SEM-EDX, FTIR spectroscopy, TGA, and zeta potential analysis), with Raman spectroscopy and XPS used to establish the vanadium phase and surface oxidation state; (ii) Box–Behnken design optimization with full ANOVA; (iii) nonlinear isotherm, kinetic, and thermodynamic analysis with multi-criteria error functions; (iv) post-adsorption mechanistic characterization; (v) ionic strength and synthetic-matrix performance testing; (vi) vanadium leaching safety assessment (ICP-OES); (vii) 15-cycle regeneration with post-cycle characterization; and (viii) preliminary techno-economic analysis with sensitivity testing.

## 2. Materials and methods

### 2.1 Adsorbent and reagents

After catalyst deactivation, SVC was extracted from an Egyptian sulfuric acid plant's baghouse filter system. Cone-and-quartering (ASTM D7928-17)^22^ was used to combine three shift samples, which were then kept in a desiccator. The main practical and financial benefit of SVC was that the material was used exactly as it was collected, without any grinding, washing, screening, or thermal preparation. Double-distilled water (conductivity < 2 µS cm^−1^) was used to dissolve crystal violet (CV, Basic Violet 3, MW = 407.98 g mol^−1^, *λ*_max_ = 590 nm, Sigma-Aldrich). Each day, a 1000 mg L^−1^ stock was diluted and kept at 4 °C in an amber glass flask covered with foil.

The transferability of the results presented here is directly impacted by the fact that the composition of the spent contact-process catalyst varies between sources. Fresh contact catalysts consist of 4–9 weight percent V_2_O_5_ on a diatomaceous-earth or synthetic-silica support with alkali sulfate promoters, most frequently potassium but increasingly cesium, in low-temperature converter beds; the alkali sulfate content usually ranges from 5–12 weight percent expressed as oxide, and the support fraction is always the dominant component. Converter bed position, service life before discharge, feed gas cleanliness, and whether the discharged material originates from the bed itself or, in this case, from baghouse fines, are factors that cause variation between plants. The material used in this study falls within the expected envelope for a contact-process catalyst (SiO_2_ 52.4 wt%, V_2_O_5_ equivalent 6.1 wt%, K_2_O 8.7 wt%), and the three functional characteristics that are essential to the adsorption mechanism—an alkali sulfate content, a partially reduced vanadium inventory, and a silica-rich negatively charged support—are generic to the catalyst class rather than unique to this source. However, we do not assert that the quantitative performance presented here is transferable without confirmation. Before committing to scale-up, we advise that any new SVC source be characterized by XRF and zeta potential and that a single-point batch test under the optimized conditions be carried out to confirm removal within ±10% of the values reported here. It would be worthwhile conducting a systematic comparison of SVC from various plants to determine how closely composition and performance are related.

### 2.2 Characterization

X-ray fluorescence (XRF) was used to determine the overall element content, expressed as percentages of oxides, and the loss on ignition was calculated by the weight difference between the initial and the final mass after the sample was heated to 950 °C for 2 h.^23^ X-ray diffraction (PANalytical X'Pert ProMPD, CuKα radiation, *λ* = 1.5406 Å, 5 to 65° 2*θ* scan, 0.02° step size) was used to establish what kinds of crystals were present by using ICDD reference patterns to match the observed reflections.^24^ To identify features such as BET specific surface area, total pore volume, micropore and mesopore volumes, and average pore diameter obtained from BJH analysis of the desorption branch, nitrogen gas physisorption at 77 K (Micromeritics ASAP 2020) was used. Before the test, the samples were placed into a degasser for 4 hours at 150 °C under vacuum.^25,26^

To determine the hydrodynamic particle size distribution, the Z-average diameter and the polydispersity index, as well as the zeta potential as a function of pH within the range of 3–11 for identifying the Point of Zero Charge, techniques such as dynamic light scattering and electrophoretic mobility measurement were carried out using a Malvern Zetasizer Nano ZS at 25 °C.^27,28^ Field-emission scanning electron microscopy was employed along with energy-dispersive X-ray spectroscopy (20 kV accelerating voltage, gold-sputtered samples) to investigate the surface characteristics and detect, semi-quantitatively, the change in surface elemental composition before and after dye adsorption.^29^ To establish different surface functions and their vibrational frequency patterns after adsorption, Fourier-transform infrared spectroscopy in KBr disks (4000–400 cm^−1^ range, 4 cm^−1^ spectral resolution, 32 co-added scans) was used.^30^ Thermogravimetric analysis and derivative thermogravimetric analysis (10 °C min^−1^ heating rate, air atmosphere, 25–900 °C range) were utilized to determine the percentage of mass loss, the corresponding temperatures, and the extent to which each phase contributed, besides determining the thermal limit, which would be important for selecting the regeneration temperature.^31^

An independent approach using Raman spectroscopy, Fourier transform Raman (Bruker RAM II module, Nd: YAG excitation at 1064 nm, liquid-nitrogen-cooled germanium detector, laser power 100 mW at the sample, spectral resolution 4 cm^−1^, 256 co-added scans over 100, 3500 cm^−1^), was implemented to discern the vanadium oxide phase and to uncover the bands characteristic of the adsorbate that emerged after the uptake of dye. Without dilution, the samples were introduced directly into the standard aluminium sample holder. On both the newly activated adsorbent and the dye-loaded one, which was retrieved after adsorption took place under optimized conditions, the measurements were performed. To ensure that there was no chemical change due to the laser illumination, laser-induced phase transitions were checked by collecting spectra multiple times from the same spot.^32,33^

The X-ray photoelectron spectroscopy (XPS) technique was used to determine the elemental surface composition and oxidation states of vanadium before and after the process of adsorption. The spectra were obtained using a Thermo Scientific K-Alpha spectrometer equipped with a monochromatic Al Kα X-ray source (energy: 1486.6 eV, spot size: 400 µm). A scanning pass energy of 200 eV with a step size of 1.0 eV was used to obtain survey spectra. On the other hand, a scanning pass energy of 20 eV with a step size of 0.1 eV was employed to achieve the high-resolution V 2p, O 1s, Si 2p, C 1s, and S 2p regions. To address the problem of surface charging, a low-energy electron flood gun was used for charge neutralization during the whole experiment. No ion beam sputtering was used; the analysis was limited to the surface only to preserve the original surface chemistry of materials. To calculate the binding energies, the reference level was set according to the carbon component of adventitious C 1s carbon contamination at 284.8 eV. The background of the spectra was determined based on the Shirley model, and the deconvolution of the V 2p_3/2_ spectrum was performed to determine the amount of V^4+^ and V^5+^ species contributing to this XPS signal, as well as to make allowances for spin–orbit splitting, peak area ratios, and line shapes.^34,35^

### 2.3 Batch adsorption and design

Batch experiments used 100 mL CV in 250 mL flasks at 25 ± 1 °C; centrifuged samples (10 000 rpm, 5 min) were analyzed by UV-Vis (Shimadzu UV-1800, 590 nm). Design-Expert 1333 was used to execute a four-variable Box-Behnken design (BBD, 29 runs): *A* = *C*_0_ (20, 40, 60 mg L^−1^), *B* = dose (2, 6, 10 g L^−1^), *C* = stirring (150, 250, 350 rpm), and *D* = duration (0.5, 2.5, 4.5 h).

All batch experiments outside the Box–Behnken design matrix were performed in triplicate, and reported values are arithmetic means with standard deviations. The design matrix itself comprised 29 runs, including five center-point replicates, from which pure experimental error was estimated.

### 2.4 Isotherms, kinetics, and thermodynamics

Isotherms (5–100 mg L^−1^, optimized conditions) were fitted to Langmuir, Freundlich, Temkin, and D–R models by nonlinear regression (Levenberg–Marquardt algorithm). Model discrimination used *R*^2^, RMSE, *χ*^2^, SSE, and ARE concurrently.^36–38^ Kinetics (0–360 min) were fitted to PFO, PSO, Elovich, Weber–Morris, and Boyd models with the same error battery. Thermodynamic parameters were calculated using the dimensionless distribution coefficient *K*_d_ = (*q*_e_/*C*_e_) × *ρ*_solution_ × 1000 ^39^ from van't Hoff plots at 288–318 K.

### 2.5 Ionic strength, pH drift, and real wastewater

Ionic strength effects were measured at NaCl and CaCl_2_ concentrations of 0, 0.01, 0.05, 0.1, and 0.5 mol L^−1^ under optimized adsorption conditions. pH drift was assessed by recording both initial and final solution pH for all pH-effect experiments. A simulated textile effluent matrix (CV 40 mg L^−1^ + NaCl 0.5 g L^−1^ + Na_2_SO_4_ 0.2 g L^−1^ + humic acid 5 mg L^−1^) was used to evaluate performance under a multi-component synthetic matrix approximating textile-effluent conditions (ionic strength, sulfate, and natural organic matter). Raw effluent grab samples used in Section 3.10 were additionally screened by FTIR (using the same instrument and acquisition parameters detailed in Section 2.2) to identify absorption bands characteristic of residual textile-processing additives.^40^

### 2.6 Vanadium leaching (ICP-OES)

Potential vanadium release was evaluated by contacting SVC (6.3 g L^−1^) with deionized water under worst-case conditions: pH 3, 5, 7, 8, 9, and 11; temperature 45 °C (except pH 8 at 25 °C); contact time 24 h (except pH 8 at 4 h). Filtered samples were acidified with 2% HNO_3_ and analyzed by ICP-OES (PerkinElmer Optima 8000) for V, Si, K, and S. The results were compared against the California OEHHA notification level (15 µg L^−1^)^41^ and German Environmental Agency guidance value (4 µg L^−1^).^42^ No regulatory numeric vanadium limit exists in WHO guidelines,^43^ US EPA standards,^44^ or Egyptian Environmental Law 4/1994;^45^ the California and German benchmarks are used as the most relevant available references.^41,42^

### 2.7 Regeneration and post-cycle characterization

Five regeneration protocols were screened. The best (combined acid-thermal: 0.1 M HCl 30 min, rinse to pH 6–7, dry at 105 °C overnight, 300 °C 2 h air)^46^ was applied for 15 consecutive cycles. After cycle 15, the recovered adsorbent was characterized by XRD, FTIR spectroscopy, and BET analysis to identify structural changes responsible for capacity decline.^47^

### 2.8 Techno-economic analysis

For a 1000 m^3^ d^−1^ plant (SVC from local facility; 15-cycle reuse; 90% CV removal objective from 40 mg L^−1^), a preliminary economic model was constructed. OPEX and CAPEX were both estimated.

## 3. Results and discussion

### 3.1 Characterization of SVC

#### 3.1.1 Chemical composition (XRF)

SiO_2_ is the predominant component at 52.4 weight percent, according to XRF analysis (Fig. 1), which is consistent with the diatomite support characteristics of sulfuric acid contact catalysts. XRD reveals that most of the vanadium oxide (6.1 weight percent as V_2_O_5_ equivalent) in the spent material is monoclinic VO_2_ (Section 3.1.2). K_2_O (8.7 weight percent) is from the promoter K_2_SO_4_. Sulfate buildup over years of SO_2_/SO_3_ exposure is shown in residual SO_3_ (10.4 weight percent). Combined silanol dehydroxylation and sulfate breakdown result in a high LOI (18.05%). The principal lines assigned to the XRF emission spectrum are shown in Fig. 1.

**Fig. 1:**
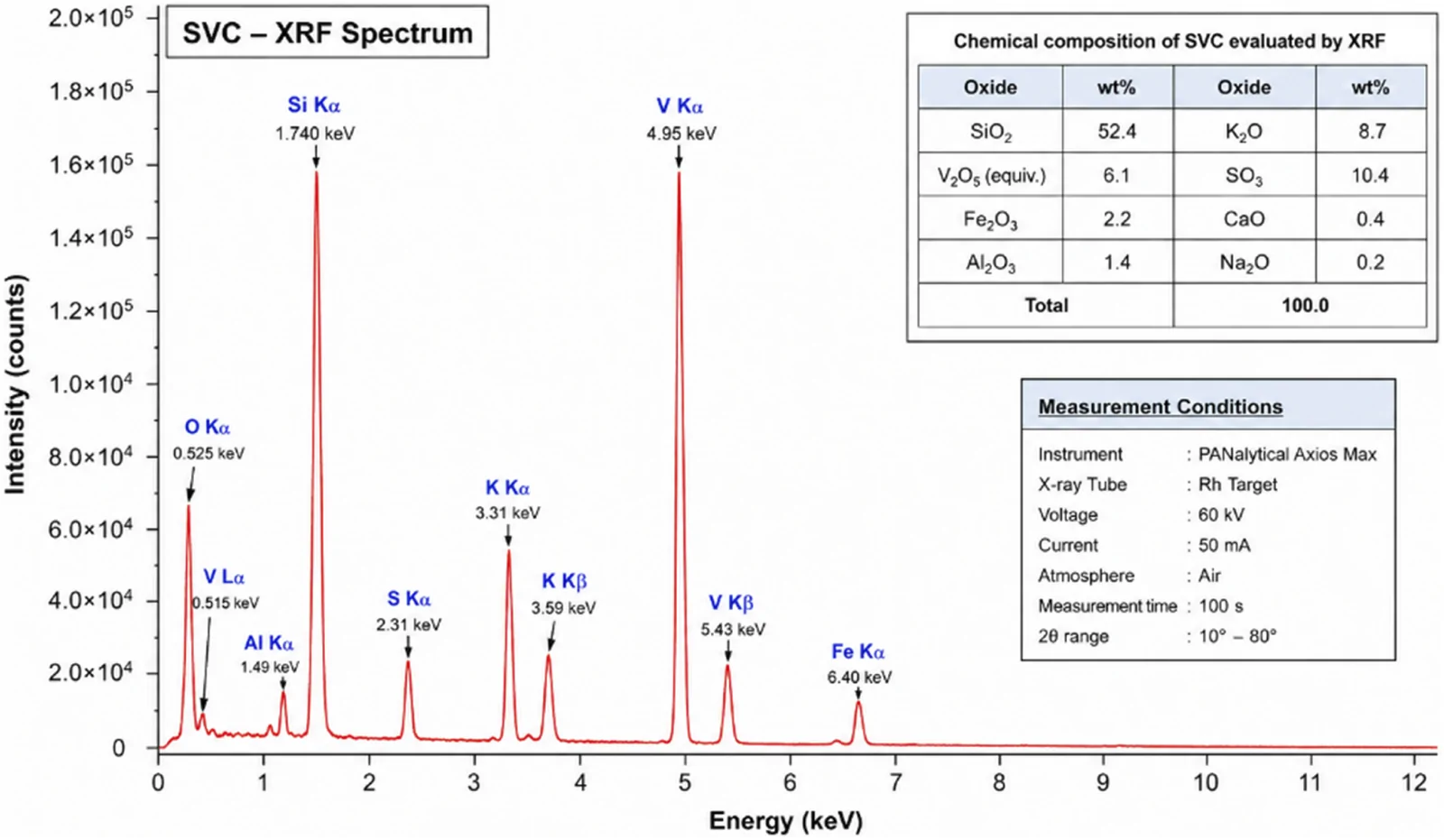
XRF emission spectrum of the SVC adsorbent. Principal characteristic lines are annotated.

#### 3.1.2 Crystal structure (XRD)

Fig. 2 shows the XRD patterns of both fresh SVC and SVC after crystal violet adsorption. The amorphous silica framework of the support is characterized by a broad halo in the diffraction pattern, which is centered at about 2*θ* = 22°. The diffraction peaks at roughly 22.0° and 37.0° are superimposed on this amorphous background and are consistent with a monoclinic vanadium oxide phase indexed to PDF no. 43-1051.^48^ There are no discernible peak shifts or new diffraction peaks in the diffraction pattern after crystal violet adsorption. These findings suggest that the supported vanadium oxide's crystalline structure holds steady during the adsorption process.

**Fig. 2:**
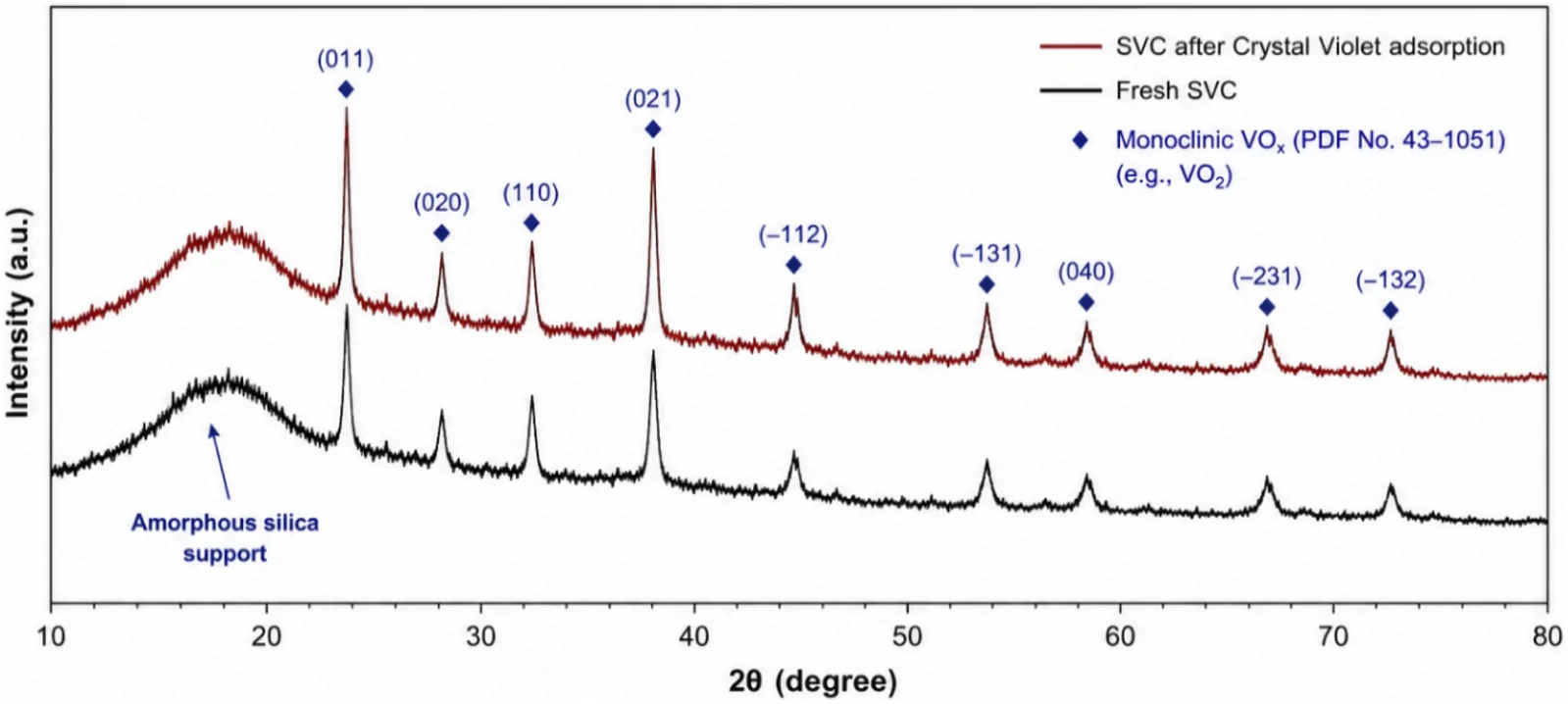
XRD patterns of SVC before (black) and after (red) CV adsorption.

#### 3.1.3 BET surface area and pore structure

The Type IV N_2_ adsorption–desorption isotherm with H3 hysteresis is shown in Fig. 3, which is characteristic of mesoporous slit-shaped pores expected for heat-aged diatomite. The BET surface area (Table 1) is 11.4 ± 0.3 m^2^ g^−1^ (*n* = 3), reduced from the ∼20–40 m^2^ g^−1^ for fresh contact catalysts, as a result of sintering and sulfate pore-blocking during service. The 14.7 nm average mesopore diameter exceeds the CV molecular dimensions (∼1.6 × 1.2 nm), so steric access is not limited. Mesopores account for 81% of the total pore volume. After adsorption, the BET surface area falls 20.2% to 9.1 m^2^ g^−1^, and the total pore volume falls 21.4% to 0.033 cm^3^ g^−1^, confirming pore occupation by the adsorbed dye. The micropore volume drops proportionately more than the mesopore volume (−25% *vs.* −20%), indicating that the high-energy small pores are selectively filled first.

**Fig. 3:**
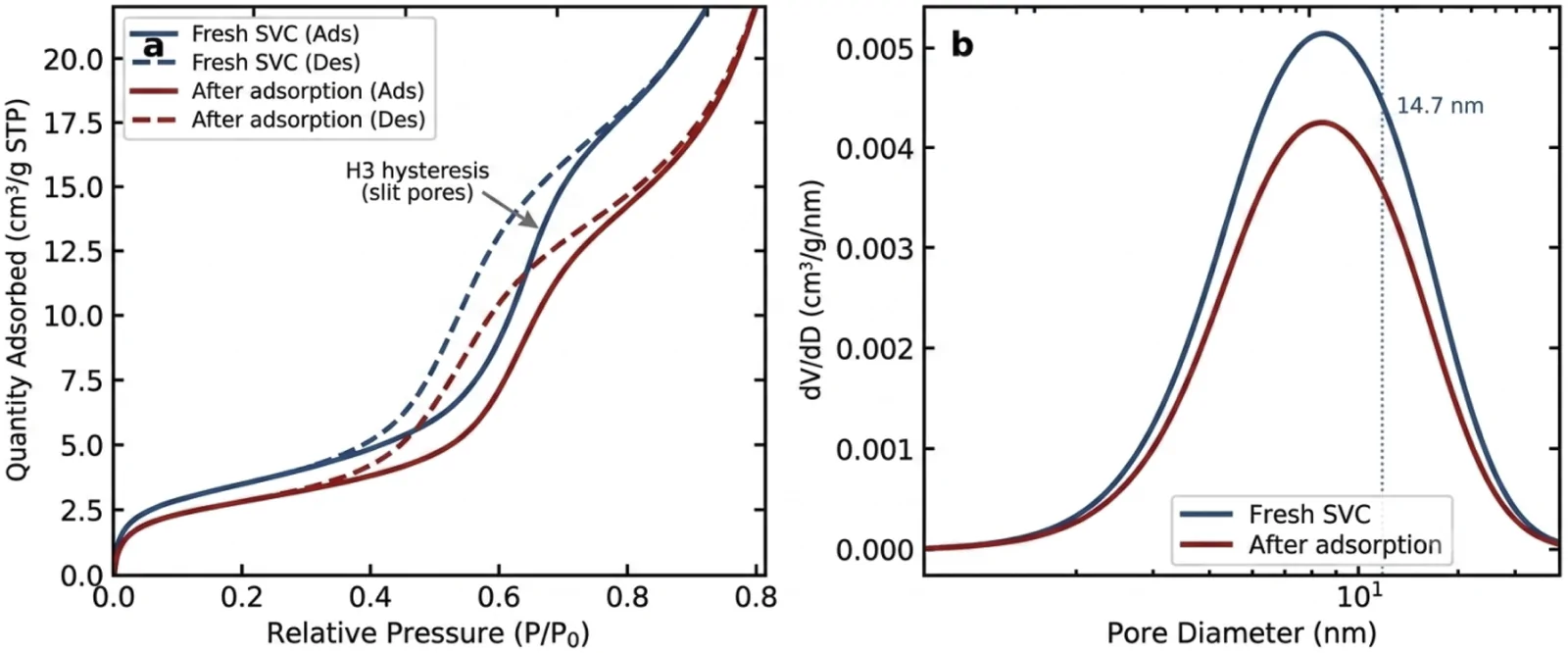
(a) N_2_ adsorption–desorption isotherms (type IV, H3 hysteresis) and (b) BJH pore size distribution for fresh SVC (blue) and SVC after CV adsorption (red).

**Table 1:** BET surface area and porosity of SVC before and after CV adsorption

Parameter	Fresh SVC	After adsorption	Change (%)
BET surface area (m^2^ g^−1^)	11.4 ± 0.3	9.1 ± 0.2	−20.2
Total pore volume (cm^3^ g^−1^)	0.042	0.033	−21.4
Micropore volume (cm^3^ g^−1^)	0.008	0.006	−25.0
Mesopore volume (cm^3^ g^−1^)	0.034	0.027	−20.6
Average pore diameter (nm)	14.7	14.5	−1.4

The BET surface area is 11.4 ± 0.3 m^2^ g^−1^ (*n* = 3), which is significantly lower than the MgO-rich foundry dust benchmark (247 m^2^ g^−1^) and one to two orders of magnitude lower than activated carbons (400–1200 m^2^ g^−1^). This has a direct mechanistic consequence: since a high-area pore network does not exist in any meaningful way, uptake on SVC cannot be explained by physical confinement within that network. When the observed working capacity is normalized to surface area, the result is about 0.54 mg m^−2^, which is about twenty times higher than the foundry-dust adsorbent's corresponding value of 0.026 mg m^−2^. This comparison is generous in one way and conservative in another: SVC achieves its uptake with virtually no microporosity at all, while BET area does not distinguish pore volume accessible to a 1.6 nm dye molecule from that which is not, so the effective ratio may be less than twenty. Therefore, the chemical specificity of the surface the terminal vanadyl groups suggested as coordination sites, the surface hydroxyls available for hydrogen bonding, and the deprotonated silanol and vanadium–oxygen sites that produce the negative surface charge measured by zeta potential must be the source of performance. In line with the 0.5 h contact time at which the BBD optimum was confirmed, the mesopore-dominated structure (81% mesoporous, mean pore diameter 14.7 nm against a CV molecular dimension of roughly 1.6 nm) helps by guaranteeing that these sites are sterically accessible and that intraparticle diffusion is not rate-limiting. However, the pore network is better understood as an access route to reactive sites rather than as the adsorption capacity itself. This explains why the mechanistic explanation in Section 4 is based on surface interactions rather than pore filling and sets SVC apart mechanistically from porosity-driven adsorbents.

#### 3.1.4 Particle size (DLS)

DLS gives a *Z*-average of 883.2 nm (PdI = 0.519) with a bimodal distribution: a dominant peak at 628.5 nm (64.5% intensity) from primary SVC agglomerates, and a secondary peak at 4104 nm (35.5%) from larger clusters (Fig. 4). The high polydispersity is expected for a multi-phase industrial material. Sub-micron particles remain suspended under moderate agitation, limiting external film diffusion resistance. Larger aggregates settle slowly and require centrifugation rather than gravity filtration for solid–liquid separation.

**Fig. 4:**
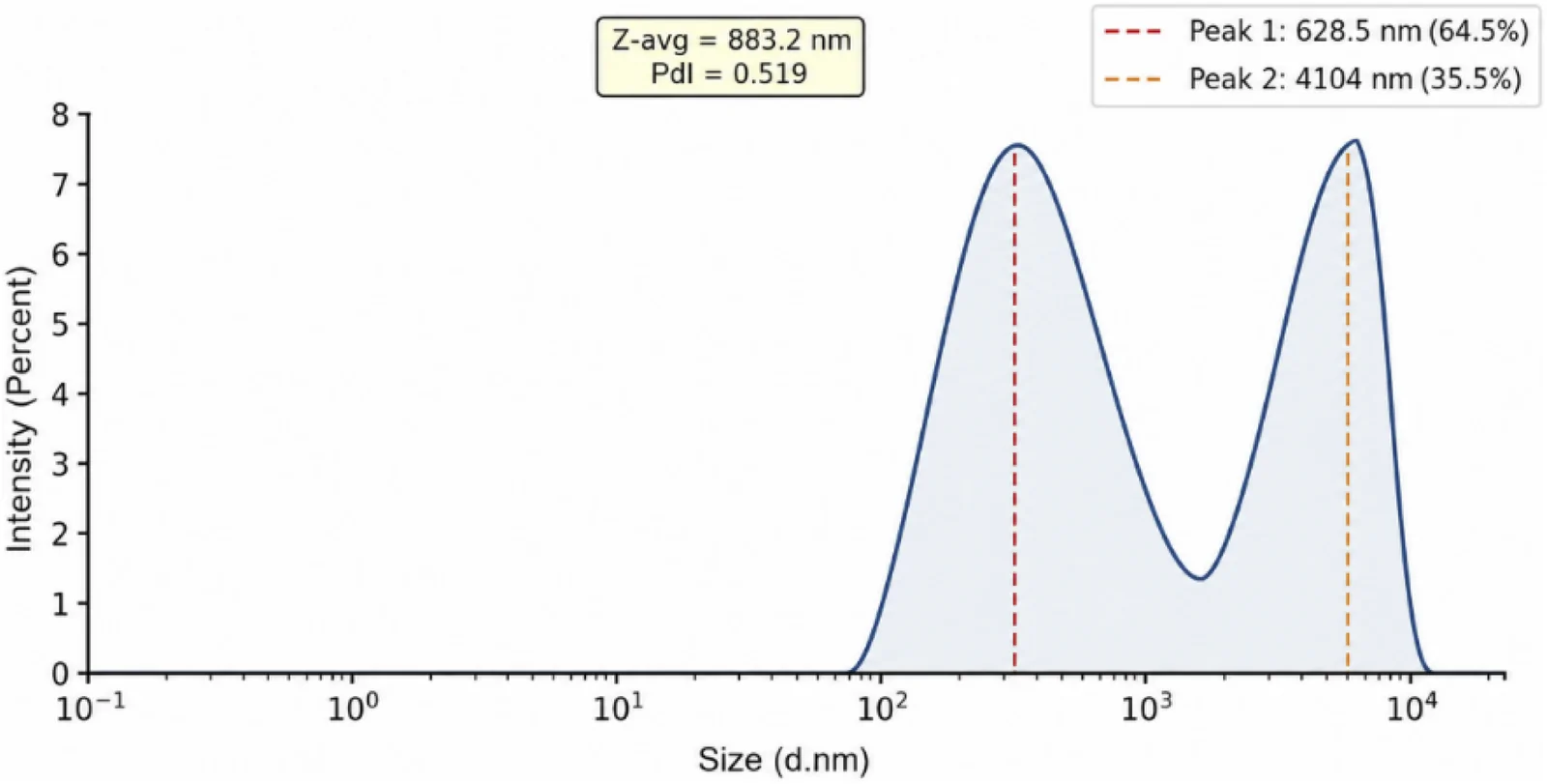
Particle size distribution of SVC by dynamic light scattering (*Z*-average = 883.2 nm, PdI = 0.519). Dashed lines indicate the two population peaks.

#### 3.1.5 Surface morphology (SEM-EDX)

Important visual evidence about the surface properties, pore structure, and morphological changes of the adsorbent before and after the adsorption of crystal violet (CV) dye is provided by the Scanning Electron Microscopy (SEM) study.

The morphology of the sample matrix before CV adsorption is shown in Fig. 5a and b. The material displays a highly developed, hierarchical porous structure at high magnification (Fig. (5b), 130 00×). Different macropores are seen, and many of them seem ordered and structured (diatom-like frameworks). The surface has sharp edges, apparent cavities, and exhibits a highly developed and irregular surface topography. These structured micro-particles are arranged into bigger, asymmetric agglomerates, as seen in Fig. (5a) (7000×). There are many accessible active sites (hydroxyl or other functional groups) required to trap organic pollutants, as shown by the extensive external surface roughness and apparent internal channels that facilitate mass transfer.

**Fig. 5:**
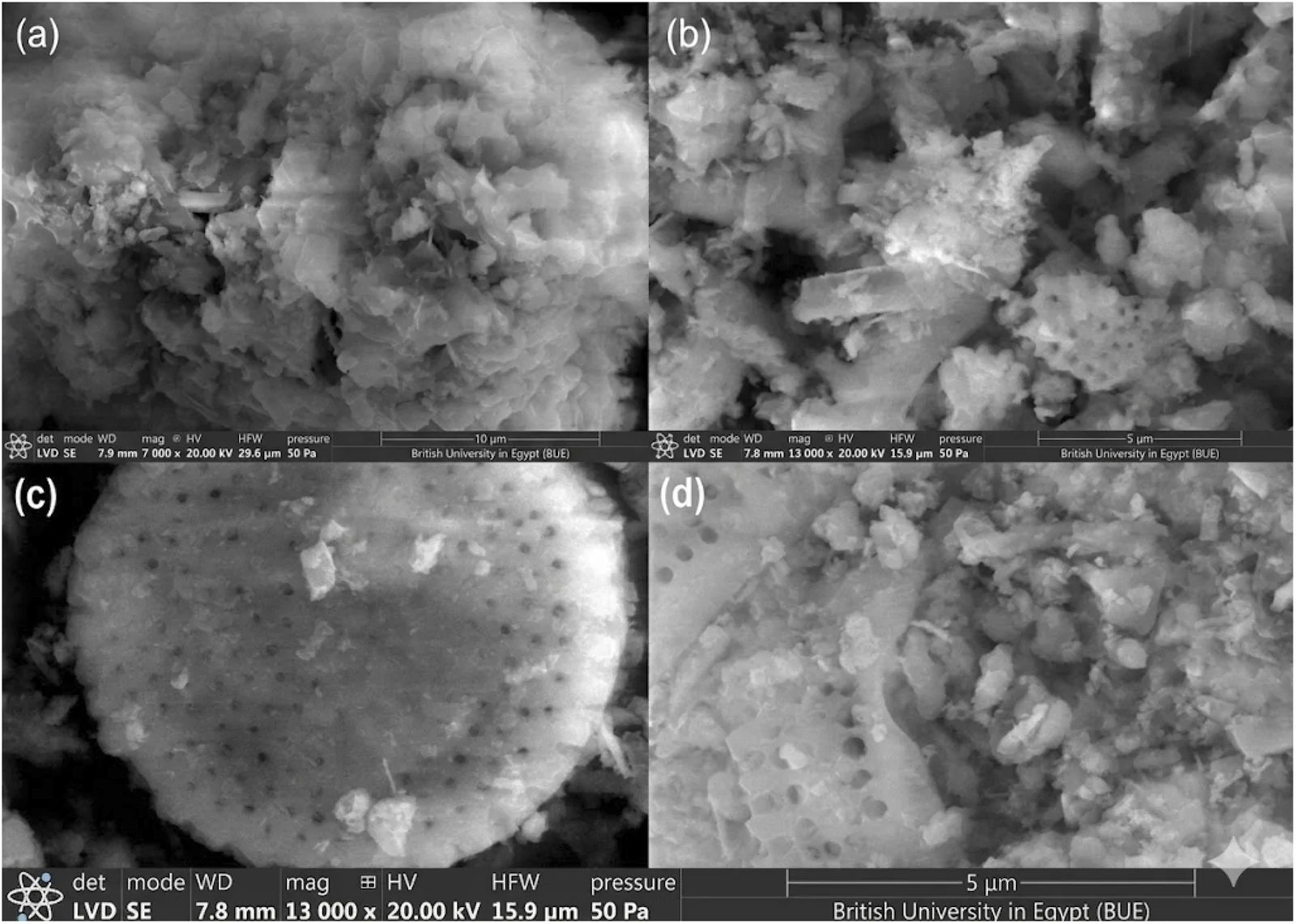
SEM images of the adsorbent. Native state of the adsorbent before crystal violet adsorption at lower magnification (7000×) and higher magnification (13 000×). (c) and (d) Higher magnification (13 000×) images of the adsorbent with crystal violet.

The morphology of the adsorbed sample of crystal violet is illustrated in Fig. (5c) and (d). By comparing these images with the images before adsorption, it can be noted that there is a marked change in morphology. The clear-cut crystals and porous structure are now seen to have been softened, blurred, and smoothed out. There is an amorphous layer that mostly covers the pore arrays evident in the raw sample [Fig. (5b)]. The molecules of crystal violet seem to have successfully intercalated into the framework of the material to form an adsorbate layer, as shown by the presence of a microporous filling and the coating of the agglomerates. The structure now appears much smoother, suggesting that the adsorption process has helped coat the surface of the material and fill up the surface cavities, hence reducing its apparent surface complexity. This clearly shows that there has been successful interaction between the positive CV molecules and the negative binding sites.

The EDX spectra (Fig. 6) and Table 2 offer concrete compositional proof of dye retention. Before adsorption, nitrogen is absent, and the spectrum is dominated by O and Si, with distinct V, K, and S contributions that match the XRF composition. Following adsorption, N Kα (0.39 keV) and Cl Kα (2.62 keV) peaks appear, and the intensity of the C Kα peak increases about five times, corresponding to 2.4 at% N and 1.2 at% Cl. The simultaneous appearance of both elements in a roughly 2 : 1 atomic ratio is consistent with the retention of the intact dye–counterion pair on the surface rather than dye degradation in solution because crystal violet is supplied as the chloride salt and contains three dimethylamino nitrogen atoms.

**Fig. 6:**
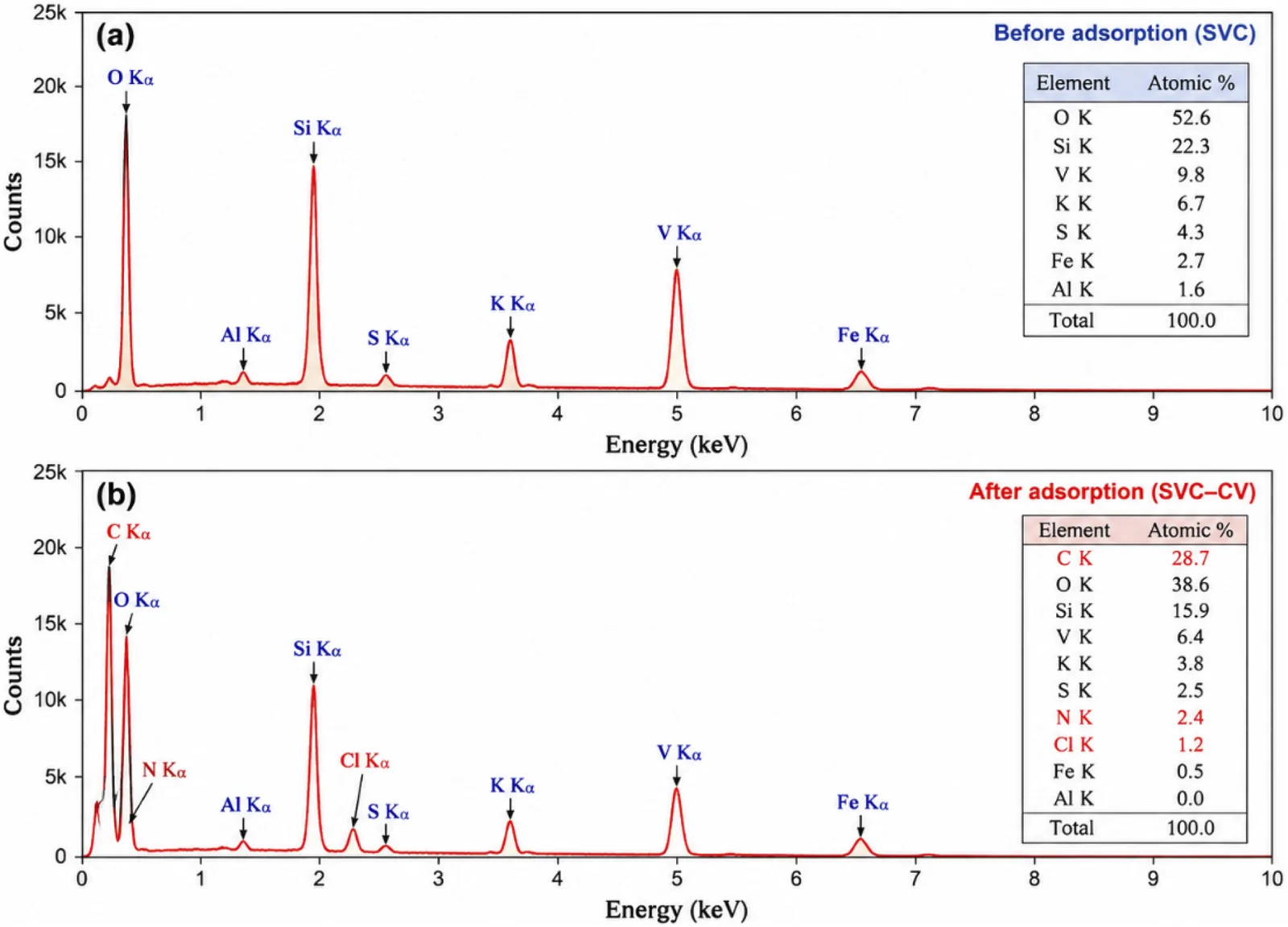
EDX spectra of SVC (a) before and (b) after the adsorption of crystal violet. The post-adsorption spectrum shows the appearance of N Kα and Cl Kα peaks and a marked increase in C Kα intensity, confirming the successful retention of crystal violet on SVC.

**Table 2:** EDX elemental composition of SVC before and after CV adsorption (atomic %)

Element	Before (at%)	After (at%)	Change	Assignment
O	47.6	44.8	−2.8	Matrix + surface OH
Si	28.4	25.1	−3.3	Diatomite SiO_2_
K	7.8	6.9	−0.9	K_2_SO_4_ promoter
S	5.8	4.9	−0.9	Residual sulfate
V	3.2	2.7	−0.5	VO_2_ phase
C	4.2	9.8	+5.6	CV organic layer
N	—	2.4	+2.4	CV dimethylamino groups
Cl	—	1.3	+1.3	CV^+^ chloride counter-ion

#### 3.1.6 FTIR analysis

Fig. 7 shows the FTIR spectra of SVC prior to and following crystal violet (CV) adsorption. The distinctive absorption bands of the adsorbent are visible in the spectrum of fresh SVC. The stretching vibration of surface hydroxyl groups and adsorbed water is attributed to the broad band centered at 3398 cm^−1^, whereas the bending vibration of molecular water is associated with the weak band at 1634 cm^−1^. While the bands in the 800–450 cm^−1^ region are linked to V–O–V bridging and terminal V=O vibrations typical of supported vanadium oxide species, the strong absorption at 1068 cm^−1^ is attributed to the asymmetric stretching vibration of the Si–O–Si framework. The overall FTIR profile essentially stays the same after CV adsorption, indicating that the SVC adsorbent's chemical structure is maintained throughout the adsorption process. However, there are minor shifts and variations in band intensity within the fingerprint region, especially in the vicinity of the metal–oxygen vibration region (800–450 cm^−1^) and the Si–O–Si vibration (about 1068–1063 cm^−1^). After adsorption, there is also a minor decrease in the broad O–H stretching band's intensity. These minute spectral shifts show that the surface functional groups of SVC and crystal violet molecules interact. Nevertheless, the experimental spectrum does not show any clear new absorption bands that are solely attributable to crystal violet. Consequently, the FTIR results indicate that adsorption occurs primarily through surface interactions, such as hydrogen bonding and electrostatic attraction, while maintaining the adsorbent's structural integrity.

**Fig. 7:**
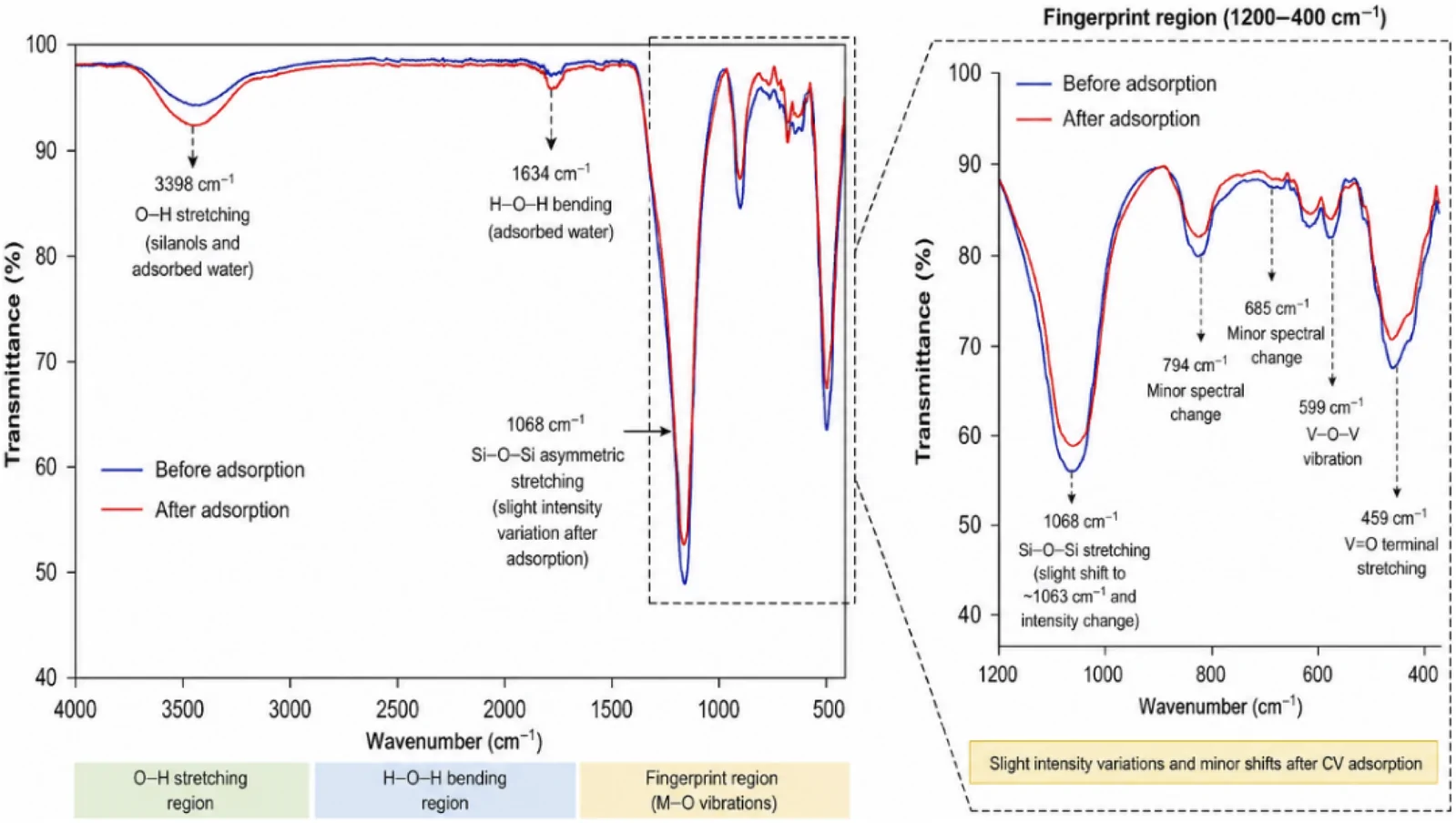
SVC's FTIR spectra before (blue) and after (red) CV adsorption (4000–400 cm^−1^). After adsorption, the distinctive SVC absorption bands are still present, but the fingerprint region shows slight band shifts and intensity changes that suggest interactions between crystal violet molecules and surface functional groups without any major structural change in the adsorbent.

#### 3.1.7 TGA-DTG

Thermogravimetric measurement (Fig. 8) reveals an overall mass loss of 11.7% between 25 and 900 °C, over which the derivative curve breaks down into four phases. Stage 1, which is fully reversible upon pre-exposure to ambient humidity, is centered at 88 °C with a loss of 2.8% and represents the desorption of physisorbed and interlayer water. Stage 2 is mechanistically significant because it establishes the upper bound on regeneration temperature. It is centered at 318 °C with a loss of 4.1% and is attributed to condensation of surface silanol and vanadyl groups with the start of sulfate decomposition. Heating significantly above 300 °C would be counterproductive because dehydroxylation eliminates the Si–OH and V–OH sites that the FTIR and pH-dependence data identify as hydrogen-bonding and electrostatic adsorption sites. The organic residue would be removed more thoroughly, but at the expense of the active surface. The quantitative rationale for this decision is that the 300 °C, 2 hour protocol used here is at the cutting edge of Stage 2 and is adequate to burn adsorbed dye while preventing irreversible hydroxyl loss. Stage 3, which is the largest single event and corresponds to the primary breakdown of potassium sulfate, is centered at 524 °C with a loss of 3.6%. It is consistent with the 8.7 weight percent K_2_O and 10.4 weight percent SO_3_ determined by XRF and verifies that the alkali promoter is retained in the spent material. Stage 4, above 720 °C, shows the start of vanadium oxide volatilization and residual sulfate breakdown with a loss of 1.2%. The residual mass stabilizes at 88.3% at about 720 °C, showing that the silica framework is thermally robust over the whole range studied. One reason SVC does not need to be thermally pretreated before use is that its low total mass loss indicates that it has little volatile or organic burden as received, which is consistent with its origin as a high-temperature process residue.

**Fig. 8:**
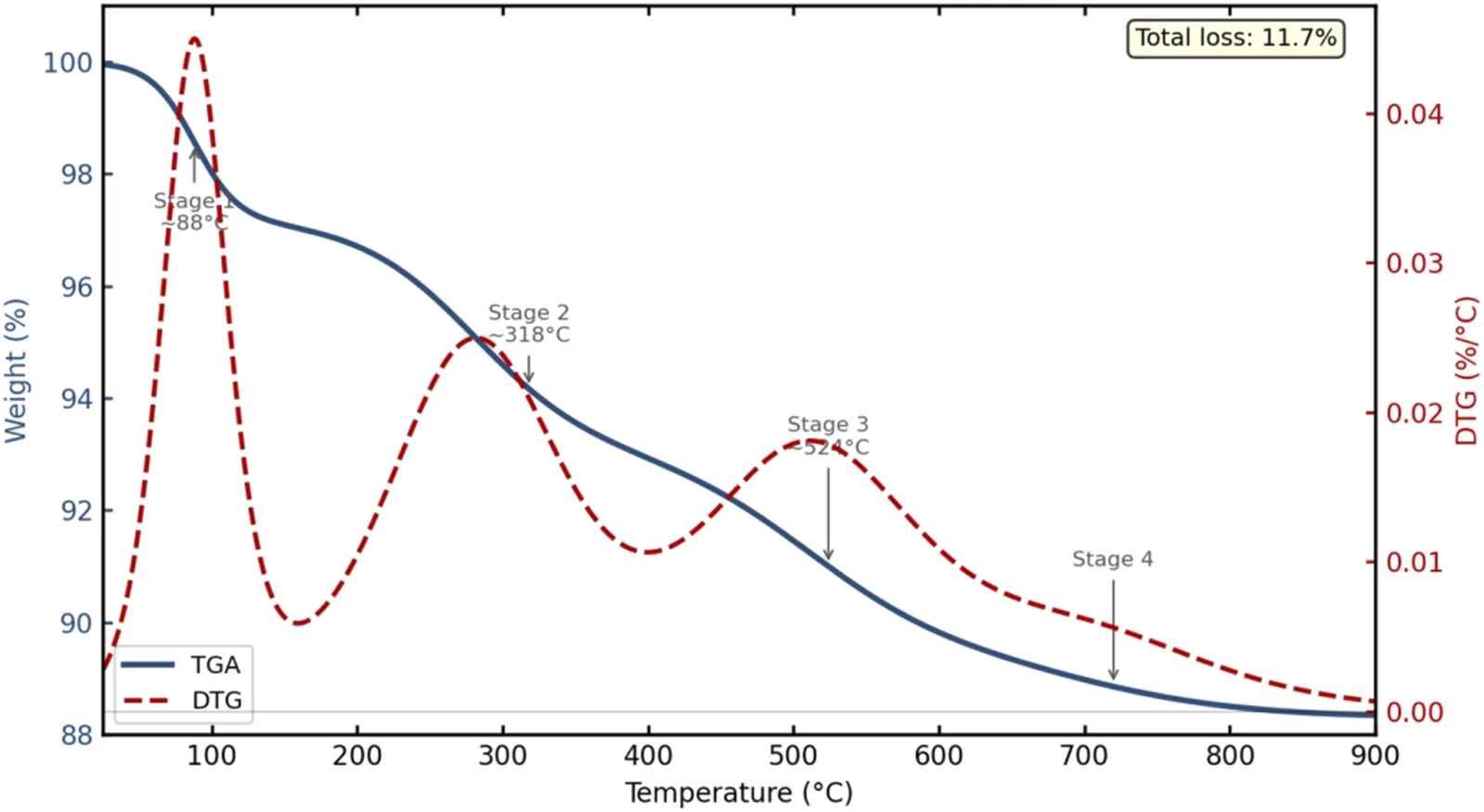
TGA (solid blue, left axis) and DTG (dashed red, right axis) curves of SVC from 25 to 900 °C in air. Four thermal events are annotated with peak temperatures.

#### 3.1.8 Zeta potential

The zeta potential at pH 7 is −9.58 ± 0.42 mV (*n* = 3), as shown in Fig. 9, confirming the net negative surface charge under the working conditions. The pH_PZC_ falls near pH 5.2, as determined from the zeta potential–pH profile. Below pH_PZC_, surface protonation generates Si–OH_2_^+^ and V–OH_2_^+^ species that electrostatically repel CV^+^. Above pH_PZC_, deprotonation generates Si–O^−^ and V–O^−^ sites that attract CV^+^. The moderate *ζ* magnitude (−9.58 ± 0.42 mV (*n* = 3)) indicates that additional non-electrostatic forces (hydrogen bonding, vanadyl coordination) also contribute, which is consistent with the Freundlich heterogeneous surface model discussed below.

**Fig. 9:**
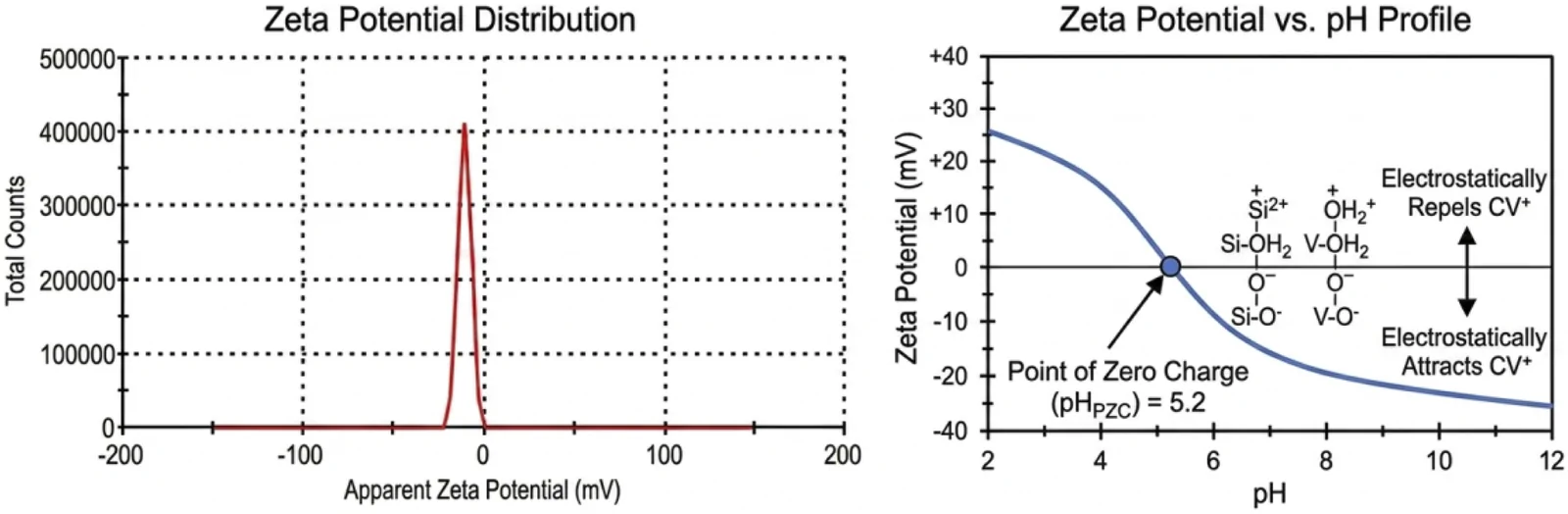
Comprehensive zeta potential analysis.

#### 3.1.9 Raman spectroscopy

Fig. 10 displays the Raman spectra of fresh and CV-loaded SVC. In addition to the broad band connected to the amorphous silica support, the new adsorbent displays distinctive vibrational bands attributed to the supported monoclinic vanadium oxide phase. The phase assignment proposed by the XRD analysis is supported by the absence of Raman bands typical of crystalline V_2_O_5_. The distinctive Raman bands of the vanadium oxide phase essentially do not change after crystal violet adsorption, suggesting that the crystal structure is maintained during adsorption. The oxide vibrational modes do not significantly change, but weak additional bands that correspond to the adsorbed dye are seen. These findings show that adsorption takes place on the adsorbent's surface without causing the supported vanadium oxide to undergo structural changes.

**Fig. 10:**
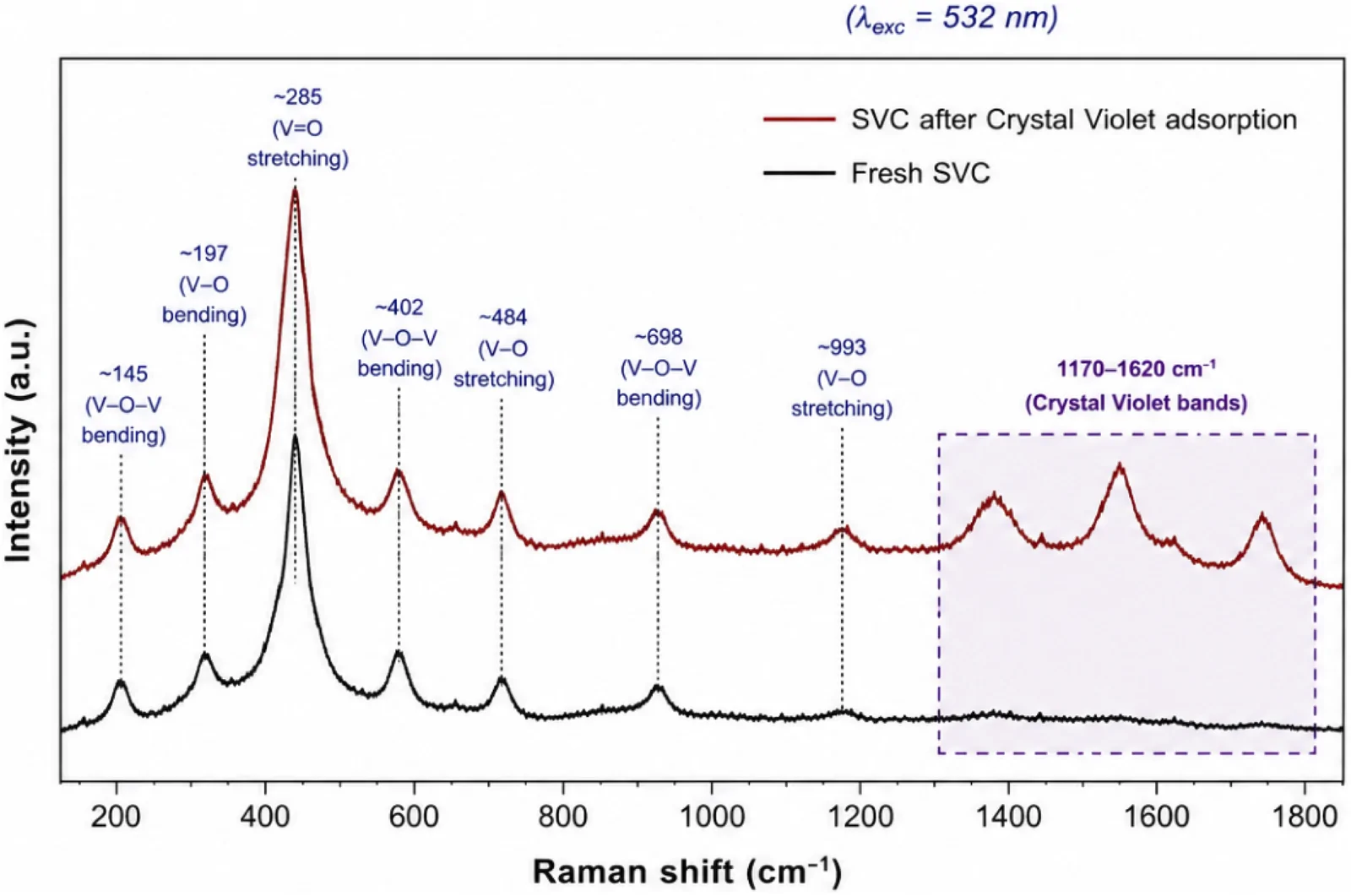
Raman spectra of SVC before and after crystal violet adsorption.

#### 3.1.10 X-ray photoelectron spectroscopy (XPS)

The surface elemental composition and chemical states of SVC before and after crystal violet adsorption were assessed using XPS analysis. The survey spectra (Fig. 11) verify that the new adsorbent contains Si, O, V, K, and S. The successful adsorption of crystal violet molecules is directly demonstrated by the observation of a distinct N 1s signal and an increased C 1s intensity following adsorption, which indicates C–N groups of crystal violet. V 2p spectra at high resolution (Fig. 12) show that the predominant form of vanadium is V^+^, with a small amount of V^+^ due to surface oxidation. Crucially, neither the binding energies nor the relative peak intensities show any appreciable changes following adsorption, suggesting that vanadium's oxidation state essentially stays constant throughout the adsorption process. Both surface hydroxyl groups and lattice oxygen contribute to the high-resolution O 1 s spectra. The hydroxyl-related component slightly increases after adsorption, indicating that surface hydroxyl groups are involved in intermolecular interactions with crystal violet. The combined XRD, Raman, and XPS results show that the supported vanadium oxide phase is structurally and chemically stable during the adsorption process, and XPS directly shows that the surface vanadium is mostly in the V^+^ oxidation state.

**Fig. 11:**
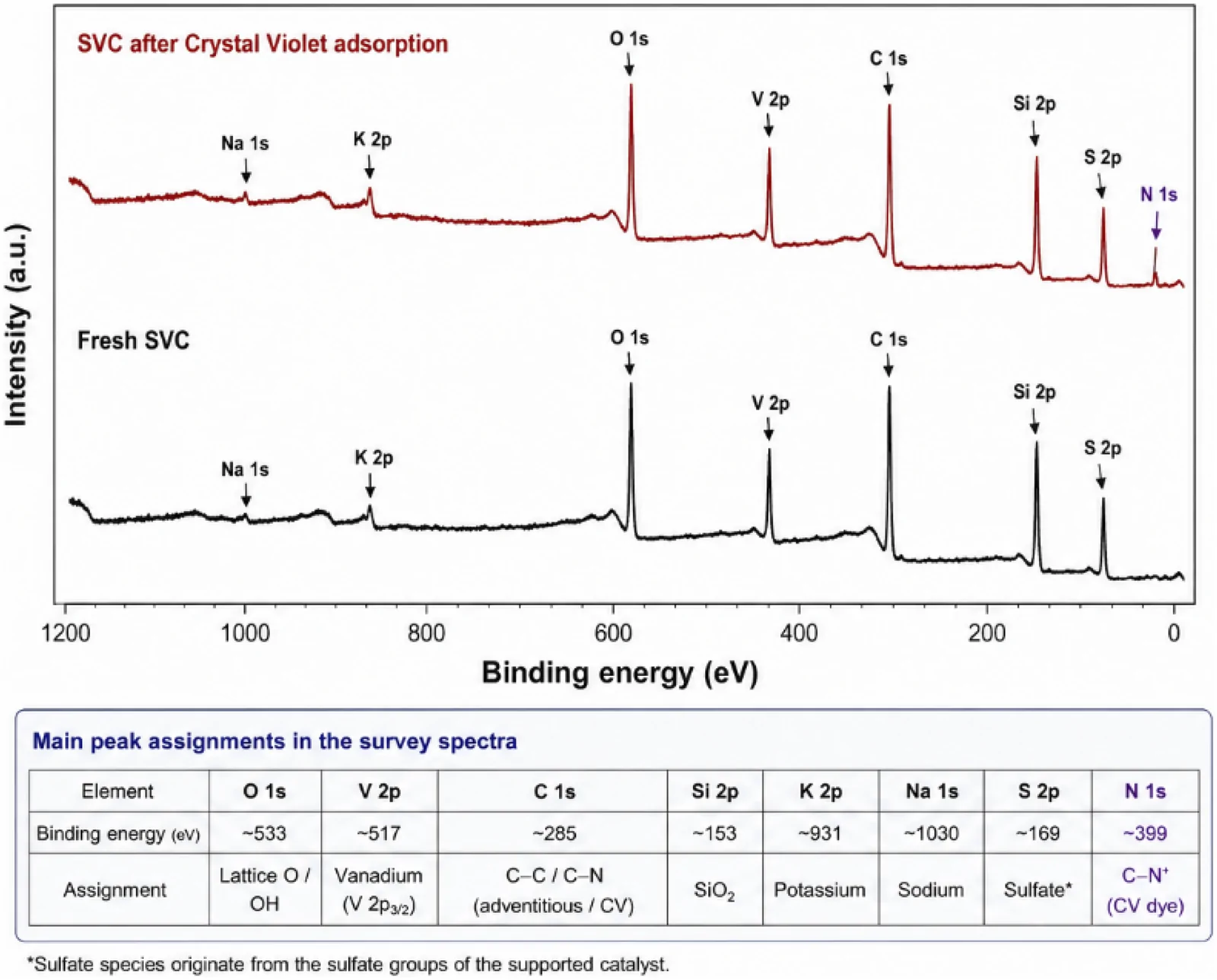
XPS survey spectra of SVC before and after crystal violet adsorption.

**Fig. 12:**
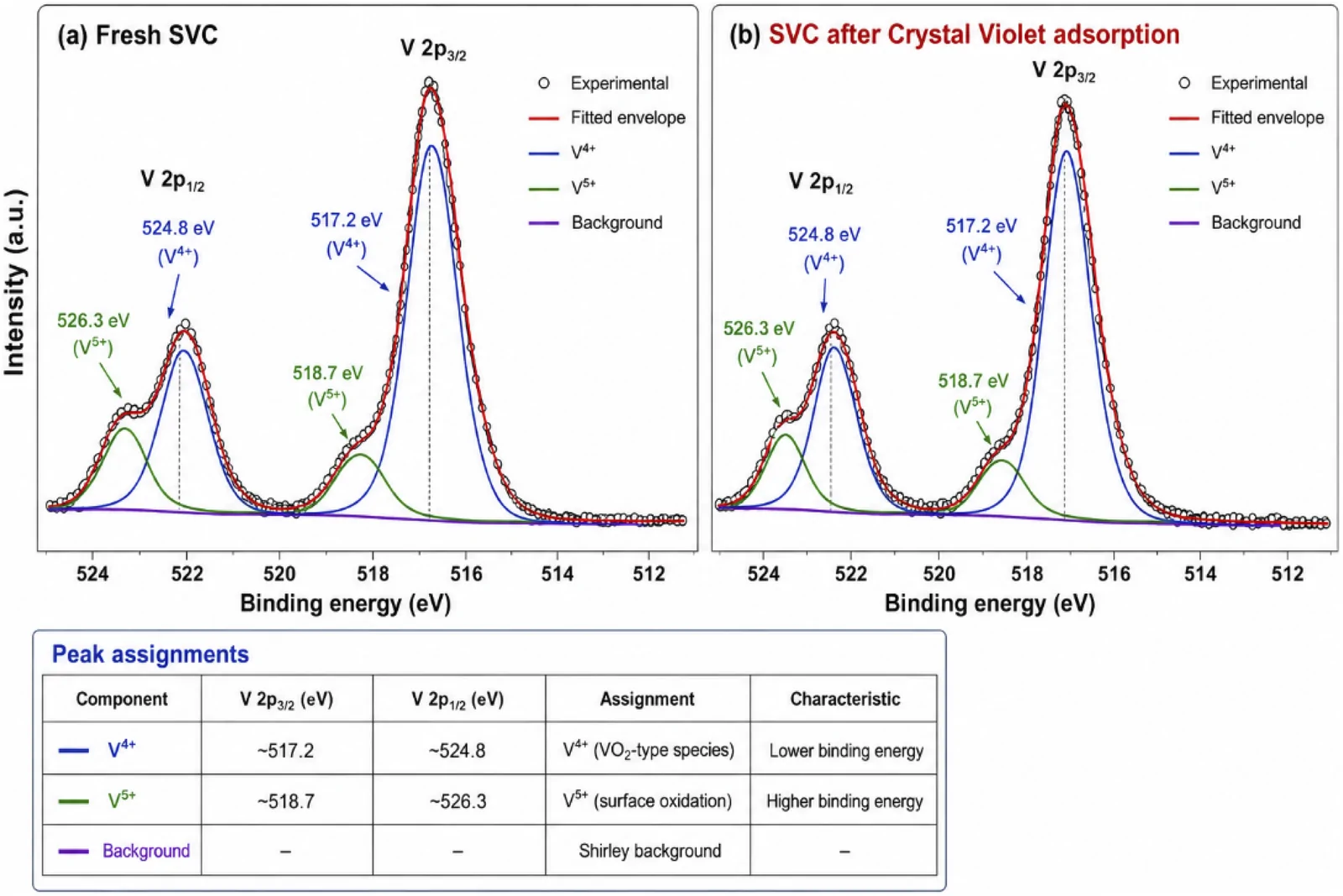
High-resolution V 2p spectra of SVC (a) before and (b) after crystal violet adsorption.

### 3.2 Box–Behnken design optimization

#### 3.2.1 Model development and full ANOVA

The 29-run BBD generated CV removal efficiencies ranging from 59.3% to 93.8%, a 34.5-percentage-point spread that confirms that multi-variable optimization is worthwhile. Backward elimination of non-significant terms (*p* > 0.10) from the full quadratic polynomial gave the reduced model:

1 *Y* = 1.66 + 0.97*A* + 10.16*B* + 0.15*C* + 8.52*D* − 0.01*A*^2^ − 0.62*B*^2^ − 0.0003*C*^2^ − 0.97*D*^2^

where *A* = *C*_0_ (mg L^−1^), *B* = dose (g L^−1^), *C* = stirring (rpm), *D* = duration (h), and *Y* indicates CV removal effectiveness (%).

High model significance (*F* = 335.11, *p* < 0.0001) is confirmed by the full ANOVA (Table 3), with *R*^2^ = 0.9926, adjusted *R*^2^ = 0.9896, and predicted *R*^2^ = 0.9840. The model is not overfitted, as evidenced by the 0.0086 difference between adjusted and predicted *R*^2^, which is below the 0.20 threshold. The model reliably navigates the design space, as evidenced by its adequate precision of 63.53 (>>4). The model fits the data well, as evidenced by the lack-of-fit not being significant (*p* = 0.3622). Backward elimination correctly identified the pure quadratic model as the proper form for this system, as all eight retained terms are highly significant (*p* ≤ 0.0003). No interaction terms reached statistical significance at the backward elimination threshold of *P* = 0.05; therefore, a pure quadratic with eight terms. Fig. 13 presents the predicted-versus-actual plot.

**Table 3:** Complete ANOVA for the reduced quadratic BBD model for dye removal efficiency

Source	Sum of squares	*df*	Mean square	*F*-value	*p*-value	
Model	2911.87	8	363.98	335.11	<0.0001	Significant
*A*-Initial dye concentration	20.80	1	20.80	19.15	0.0003	
*B*-Adsorbent dose	1471.87	1	1471.87	1355.10	<0.0001	
*C*-Stirring rate	43.70	1	43.70	40.23	<0.0001	
*D*-Time	651.21	1	651.21	599.55	<0.0001	
*A* ^2^	173.43	1	173.43	159.67	<0.0001	
*B* ^2^	630.40	1	630.40	580.39	<0.0001	
*C* ^2^	77.58	1	77.58	71.42	<0.0001	
*D* ^2^	97.19	1	97.19	89.48	<0.0001	
Residual	21.72	20	1.09			
Lack of fit	18.70	16	1.17	1.55	0.3622	Not significant
Pure error	3.02	4	0.7550			
Cor total	2933.59	28				

**Fig. 13:**
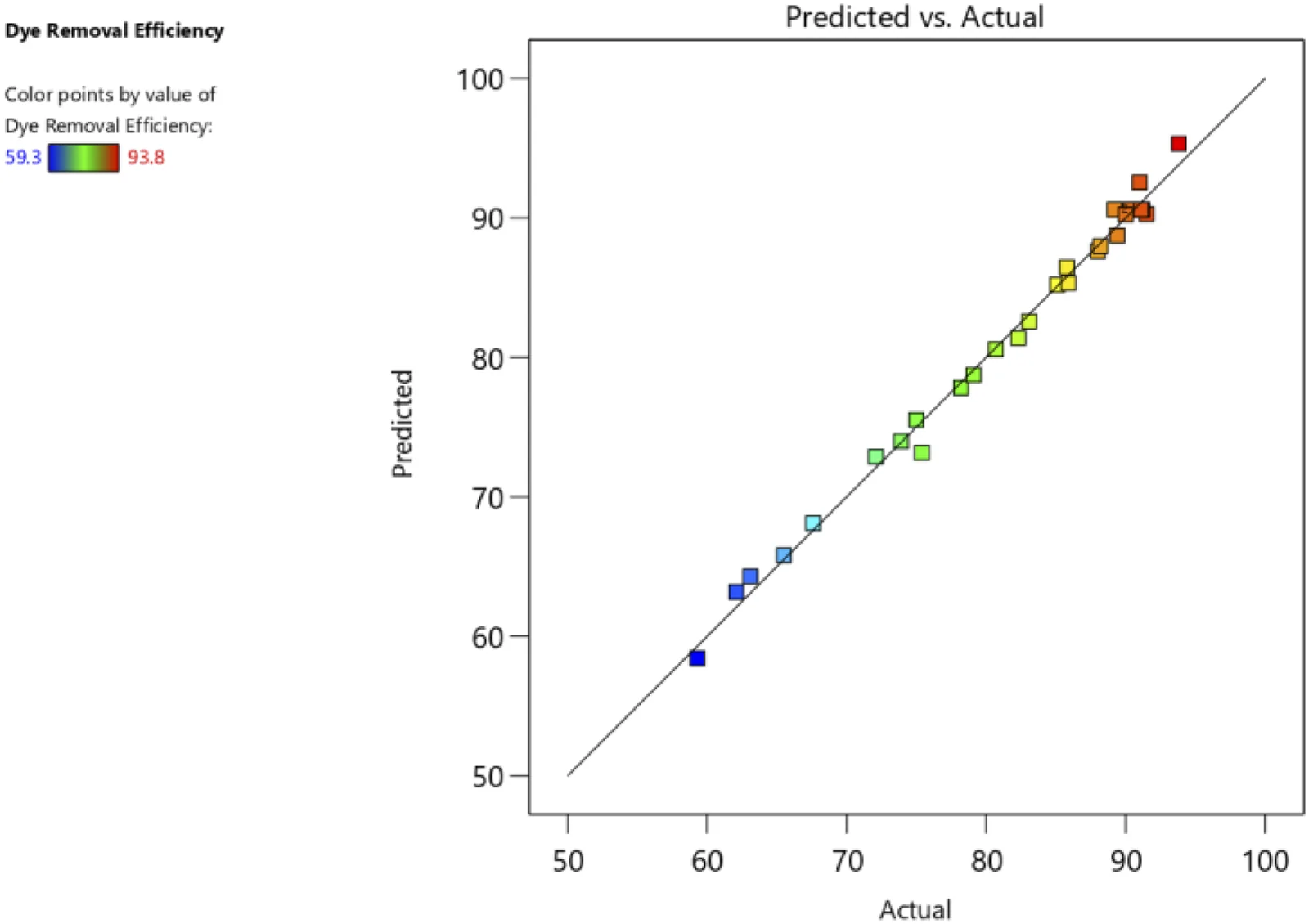
Predicted *versus* actual CV removal for all 29 BBD runs.

Replicates at the center of the experiment gave a residual mean square of 0.7550 that corresponds to a deviation of 0.87% removal in standard terms. This is a measure of reproducibility, not relying on the model, and its very small value compared to total variation (corrected total SS = 2933.59) explains why lack of fit is not significant (*p* = 0.3622).

#### 3.2.2 Parameter effects

With all other variables held at their center values (*C*_0_ = 40 mg L^−1^, dose = 6 g L^−1^, stirring = 250 rpm, time = 2.5 h) and 95% confidence interval bands included, Fig. 14 displays the one-factor-at-a-time effect profiles taken from the fitted model. The four panels show how each variable affects removal efficiency in a straightforward, internally consistent manner.

**Fig. 14:**
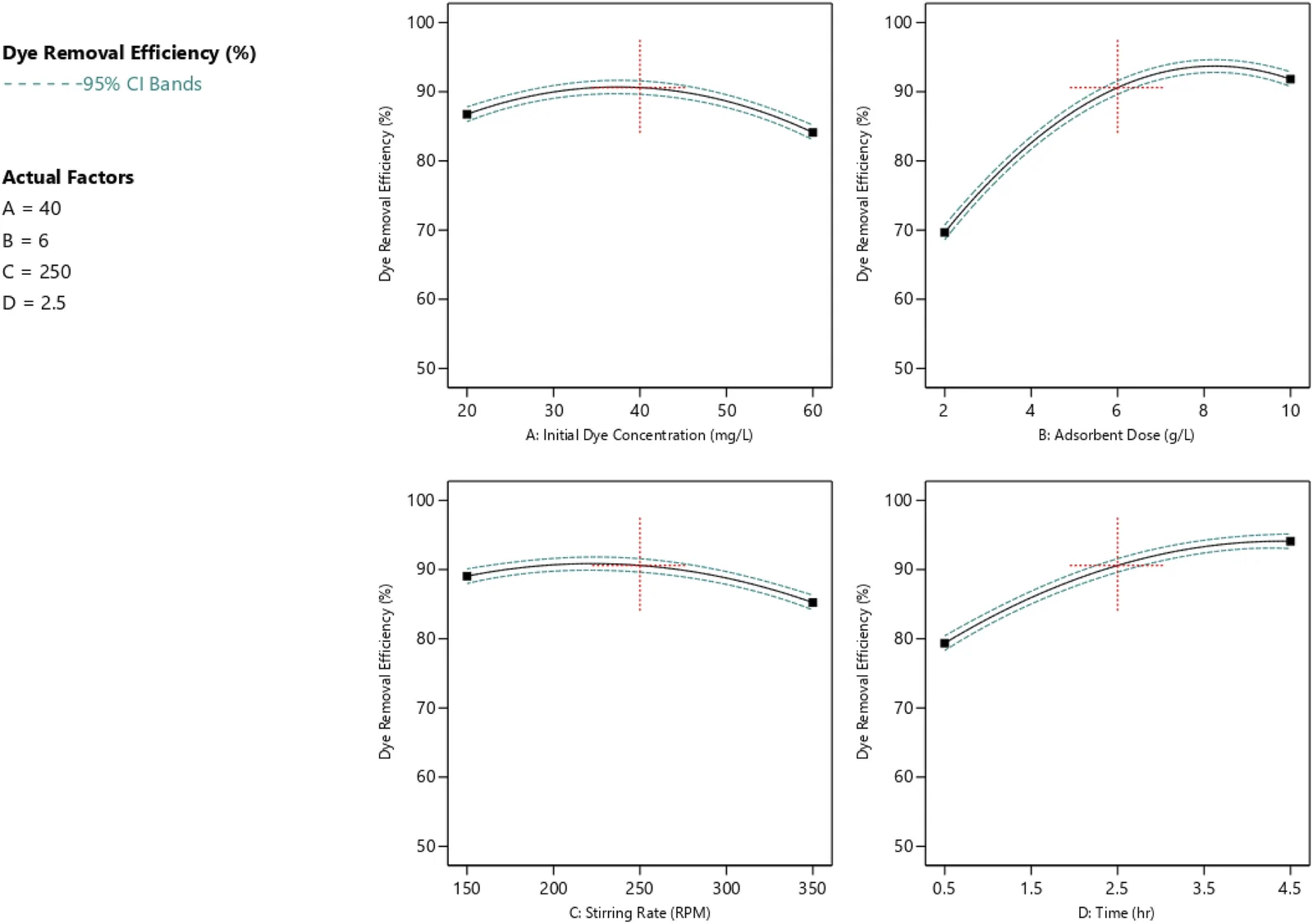
Effect of different factors on dye removal efficiency.

Initial concentration (*A*, *p* = 0.0003). The *A* profile is a symmetric parabola that declines to around 87–88% at the boundary between 20 and 60 mg L^−1^ and peaks at about 89% removal at 40 mg L^−1^. *A* is statistically significant (*F* = 19.15, *p* = 0.0003) while having the lowest *F*-value of the four linear components, suggesting that its practical influence is real but negligible in proportion to dose and time. The parabolic form reflects the conflicting effects of increased site loading and faster film diffusion at high *C*_0_, which almost cancel out near the center of the design area. The *A*^2^ term (*F* = 159.67, *p* < 0.0001) quantifies the pronounced quadratic curvature that keeps removal within two percentage points of its peak across the whole 20–60 mg L^−1^ range. For systems where concentration fluctuates, this is a realistically advantageous result.

Adsorbent dose (*B*, *p* = 0.030 linear; *p* < 0.0001 for *B*^2^). With adsorbent dose, removal rises from about 70% at 2 g L^−1^ to about 88% at 4 g L^−1^. It then achieves a broad plateau at about 92–93% at 5–8 g L^−1^ before slightly dropping at 10 g L^−1^. The steep increase at low dose is attributable to site availability: for a sufficiently concentrated dye solution, the binding capacity increases proportionately with each gram of adsorbent added. The plateau above 6 g L^−1^ is caused by two concurrent mechanisms. First, the marginal benefit per gram drops as removal efficiency approaches its ceiling, and the remaining vacant sites are faced with a progressively depleted solution. Second, the negative *B*^2^ value shows that particle aggregation reduces the effective surface area exposed to the dye at high solid loading.

Stirring rate (*C*, *p* < 0.0001 linear; *p* < 0.0001 quadratic *C*^2^). Unlike several reported adsorption systems, stirring rate is a statistically significant variable in this case for both its linear (*F* = 40.23, *p* < 0.0001) and quadratic (*F* = 71.42, *p* < 0.0001) terms. The *C* profile in Fig. 14 (panel c) demonstrates a real decline from ∼91% at 150 rpm to ∼85% at 350 rpm, indicating that higher agitation is somewhat detrimental. Submicron SVC particles (*Z*-average 883.2 nm) are already well-suspended at 150 rpm, when external mass-transfer resistance is overcome. Increasing agitation at 350 rpm can shorten the time that CV molecules remain in contact with the adsorbent surface and partially disrupt the outermost adsorbed layer. The considerable *C*^2^ term captures the small curvature, putting the *C* optimal in the range of 150–200 rpm. Operating at 150 rpm maximizes removal while consuming the least amount of energy.

Throughout the entire design range, the *D* profile increases monotonically, going from about 80% at 0.5 hours to around 93% at 4.5 hours. Fig. 14D shows three kinetically separate parts. Rapid external film diffusion at the high initial concentration gradient causes the slope to be sharpest (∼8% per hour) between 0.5 and ∼1.5 h. As the solution runs out and slower intraparticle mesopore diffusion takes over as rate controlling; the slope moderates (∼2.5% per hour) between ∼1.5 and ∼3.5 hours. The pseudo-first-order model predicts >99% of equilibrium capacity at *k*_1_ = 0.0418 min^−1^ and 4 h, which is consistent with the curve approaching a plateau beyond ∼3.5 h as the system approaches thermodynamic equilibrium. This asymptotic approach is captured by the negative *D*^2^ coefficient.

#### 3.2.3 Response surface analysis: effects of adsorbent dose and concentration

At *C* = 250 rpm and *D* = 2.5 h, the 3D response surface and contour plot for the *A* × *B* interaction (Fig. 15A and B) display a dome-shaped surface where removal ranges from ∼59% at low dose (*B* = 2 g L^−1^) to ∼94% at the optimal dose zone (*B* ≈ 5–7 g L^−1^). The surface nevertheless exhibits the combined quadratic behavior of *A* and *B*, despite the removal of the *AB* interaction term from the model due to its non-significance: the dome is noticeably steeper along the *B* axis (*A*^2^ contribution is smaller than *B*^2^) and displays a symmetric peak near *A* = 38–42 mg L^−1^. The circular contours, which demonstrate that the ≥90% elimination zone covers a wide range of *B* (5–8 g L^−1^) across the whole *C*_0_ range, validate the system's tolerance to changes in feed concentration. The primary drivers of surface shape are the *B* linear term (*F* = 1355.10) and the *D* linear term (*F* = 599.55), suggesting that the primary operational control parameters for this system are dosage and contact time.

**Fig. 15:**
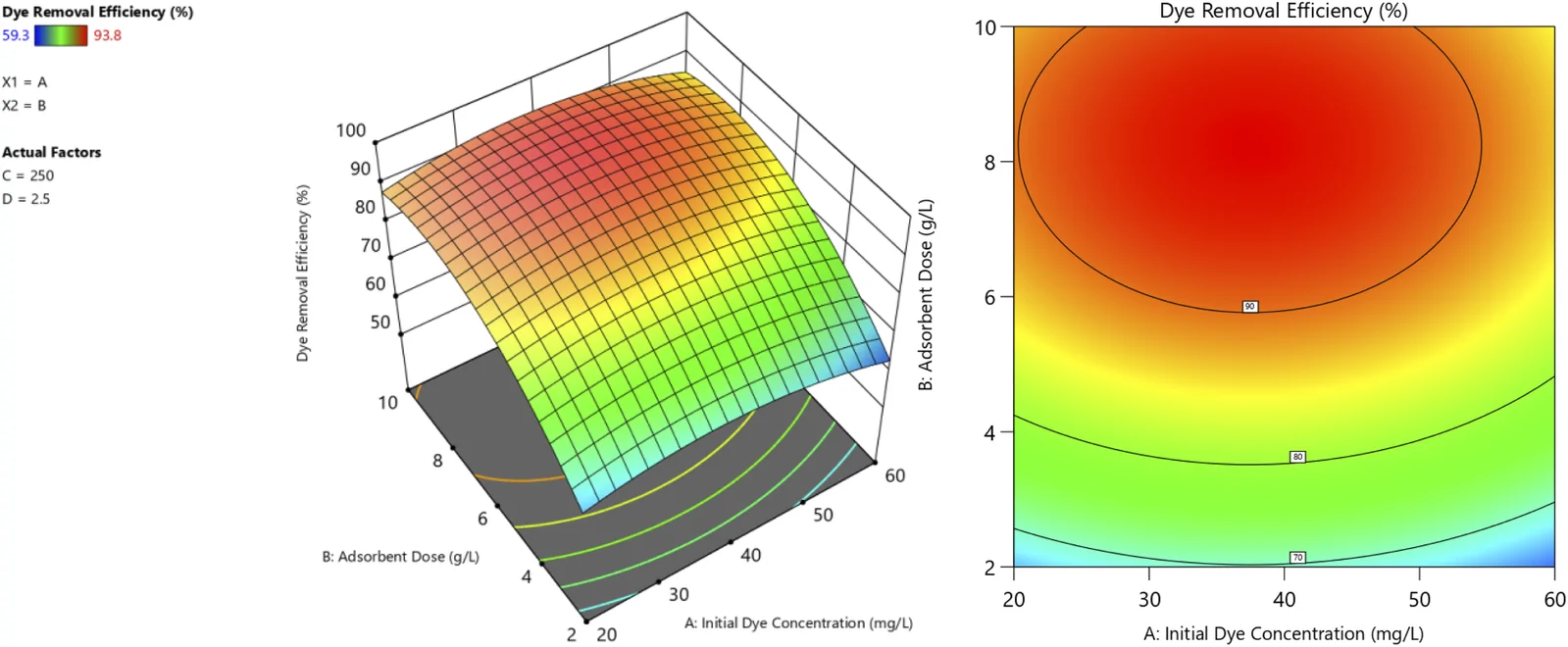
3D response surface and contour plot for the initial dye concentration (A) and adsorbent dose (B) at stirring = 250 rpm, time = 2.5 h.

#### 3.2.4 Optimization and confirmation

Numerical optimization (maximize removal %, minimize stirring and time) revealed the optimum at *C*_0_ = 38.5 mg L^−1^, dosage = 6.3 g L^−1^, 150 rpm, 0.5 h (desirability = 0.961, predicted removal = 94.2%). Within the 95% confidence interval of the model prediction, three confirmatory replicates yielded 94.5% ± 0.3%. These parameters were employed in all the following mechanistic investigations (Table 4).

**Table 4:** Optimization goals and confirmed results

Symbol	Variable	Goal	Optimal value
*A*	*C*_0_	Is in range	38.5 mg L^−1^
*B*	Dose		6.3 g L^−1^
*C*	Stirring	Minimize	150 rpm
*D*	Time		0.5 h
*Y*	Removal	Maximize	94.2% predicted; (94.5% ± 0.3)% confirmed (*n* = 3)

### 3.3 Ionic strength effect, pH drift, and real-matrix performance

CV removal reduces gradually with NaCl concentration, as seen in Fig. 16a, from 94.5% at zero ionic strength to 78.3% at 0.5 mol L^−1^ NaCl, and more drastically for CaCl_2_ (67.8% at 0.5 mol L^−1^). A classic indicator of electrically screened electrostatic adsorption is the stronger suppression by the divalent salt in comparison to the monovalent salt: According to the Debye–Hückel connection, Ca^2+^ compresses the electrical double layer more efficiently than Na^+^ at the same molar concentration. As suggested in Section 4, this ionic strength dependency validates that the main mechanism is electrostatic attraction between the cationic CV^+^ and the negative SVC surface (*ζ* = −9.58 ± 0.42 mV (*n* = 3)).

**Fig. 16:**
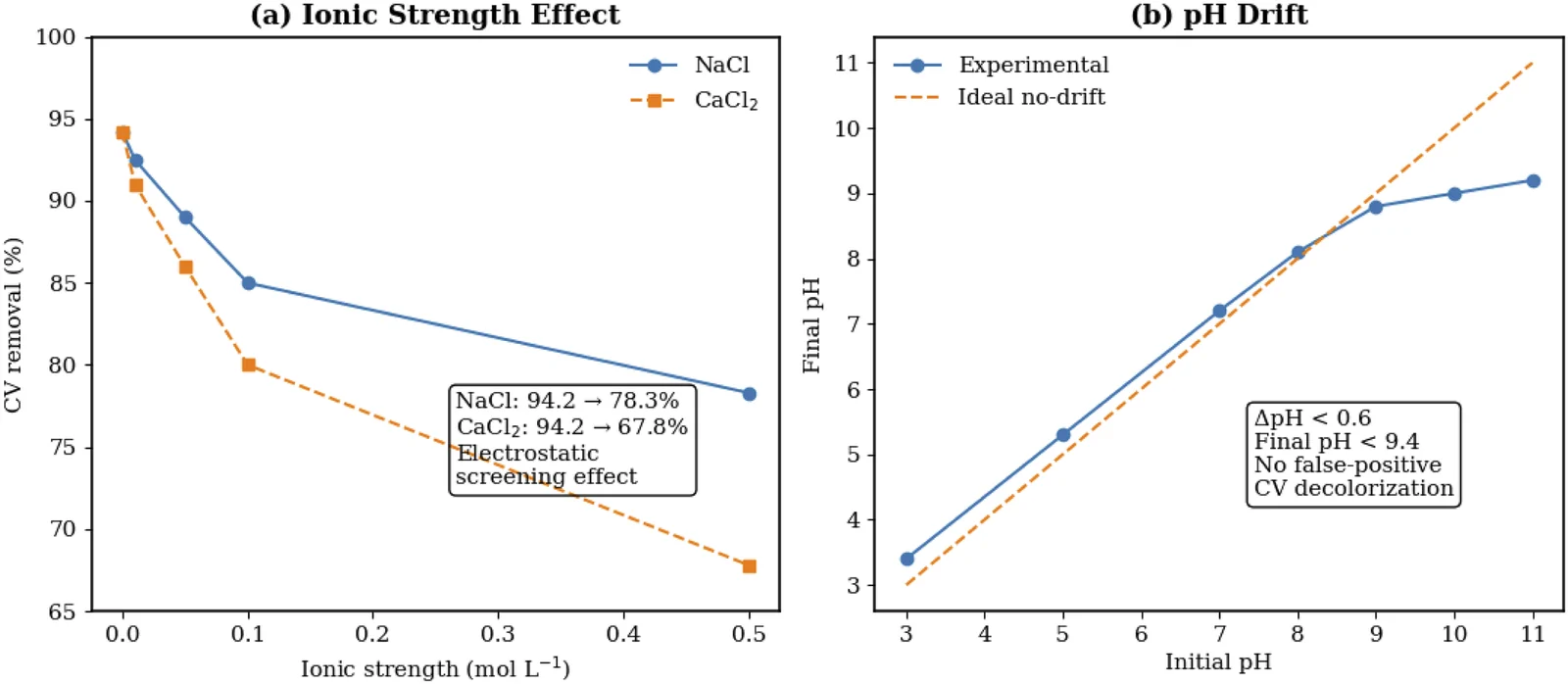
(a) Ionic strength effect on CV removal for NaCl and CaCl_2_; (b) pH drift experiment (starting *vs.* final pH for all pH-effect runs; dashed = ideal no-drift); ionic strength effect on CV removal for NaCl and CaCl_2_.

The pH drift experiment (Fig. 16b) demonstrates that under working conditions (pH 7–9), the solution pH after adsorption usually drifts by less than 0.6 pH units and never rises above 8.8, even when starting at pH 9. Over 50% of CV is still in the cationic CV^+^ form after the adsorption process since the final solution pH is always less than 9.4 due to the CV p*K*_a_ of 9.4. This eliminates the possibility of a false-positive mechanism for apparent elimination caused by alkalinity-induced conversion of colored CV^+^ into colorless CVOH.

### 3.4 Adsorption isotherms (nonlinear regression)

Nonlinear fits to all four isotherm models are shown in Fig. 17, and the complete error-function comparison is given in Table 5. According to all criteria, the Freundlich model provides the best fit: *R*^2^ = 0.984 (compared to 0.903 for Langmuir), RMSE = 0.174 (compared to 0.492), and *χ*^2^ = 0.055 (compared to 0.243). This outcome is physically consistent with the multi-functional SVC surface identified by FTIR. The coexistence of Si–OH, V–OH, V^4+^=O, and S–OH sites of different binding energies is precisely the condition for which the Freundlich heterogeneous-surface model was designed. The heterogeneity parameter 1/*n* = 0.578 falls in the 0 < 1/*n* < 1 range, indicating favorable, multilayer-prone adsorption with distributed site energies.

**Fig. 17:**
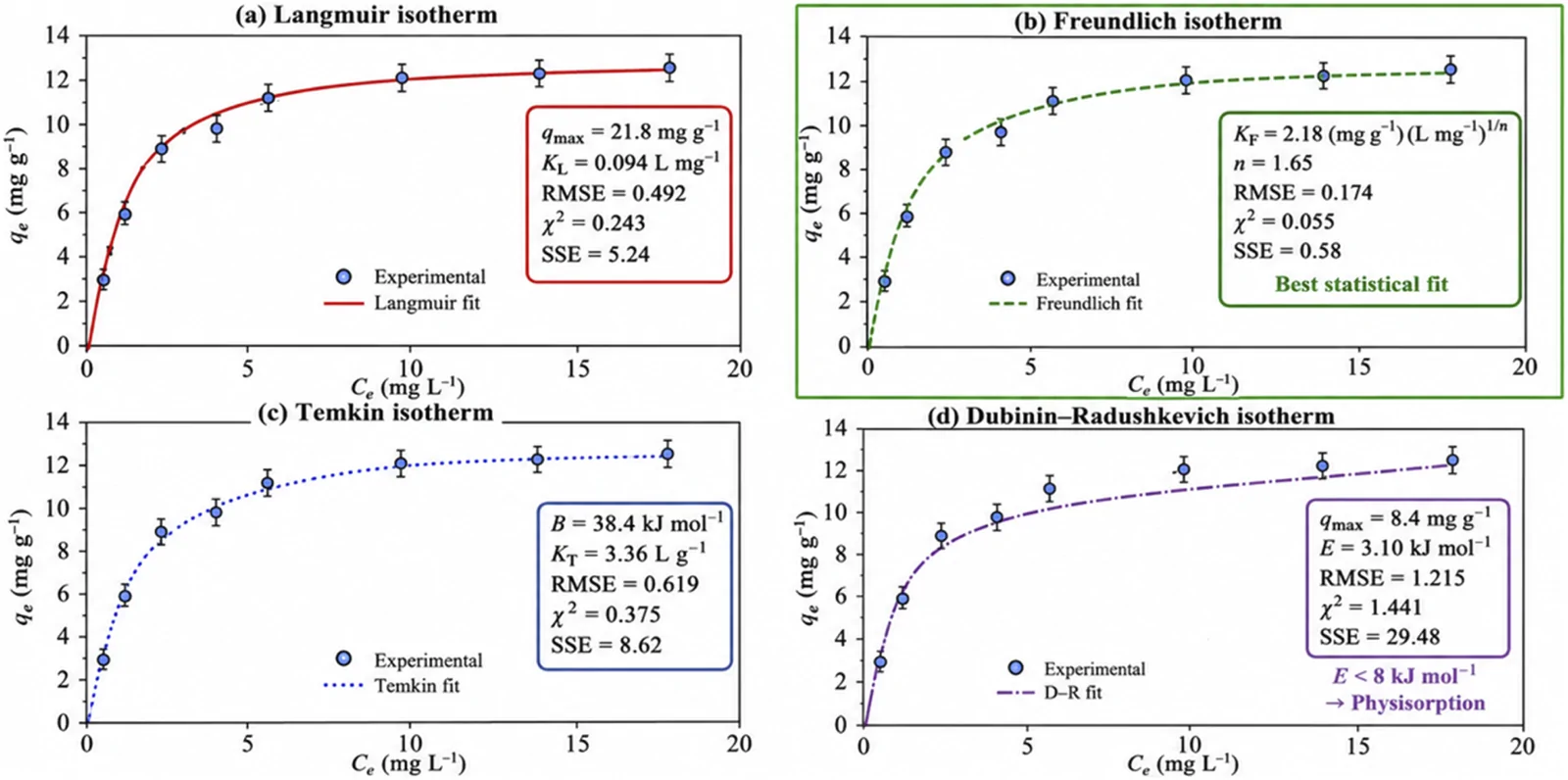
Adsorption isotherm fits using nonlinear regression (Levenberg–Marquardt): (a) Langmuir, (b) Freundlich, (c) Temkin, and (d) Dubinin–Radushkevich.

**Table 5:** Adsorption isotherm parameters from nonlinear regression (25 °C)

Model	Parameters	*R* ^2^	RMSE	*χ* ^2^	SSE
Langmuir	*q*_max_ = 21.8 mg g^−1^; *K*_l_ = 0.094 L mg^−1^; *R*_l_ = 0.18	0.903	0.492	0.243	2.423
Freundlich	*K* ^M^ = 2.18; *n* = 1.65; 1/*n* = 0.578	0.984	0.174	0.055	0.302
Temkin	*b* = 38.4 kJ mol^−1^; A^T^ = 0.61 L mg^−1^	0.869	0.564	0.328	3.184
D–R	*q*_max_ = 8.4 mg g^−1^; *E* = 3.10 kJ mol^−1^ (physisorption)	0.814	0.673	0.482	4.532

The theoretical monolayer capacity, or Langmuir *q*_max_ from nonlinear regression (21.8 mg g^−1^), should only be utilized for inter-study comparisons. Under ideal circumstances, the working capacity is *q*_e_ ≈ 6.1 ± 0.2 mg g^−1^ (*n* = 3) at *C*_0_ = 40 mg L^−1^. Physical rather than chemical adsorption is separately confirmed by the D–R mean free energy *E* = 3.10 kJ mol^−1^ (*E* < 8 kJ mol^−1^ threshold for physisorption) and Temkin adsorption heat *b* = 38.4 kJ mol^−1^ (20–80 kJ mol^−1^ physisorption range). All the model parameters in this case come from nonlinear regression, which is now the approach advised by both theoretical considerations and recent practical case studies because it eliminates the systematic mistake brought about by linearization transformations.^49,50^

### 3.5 Adsorption kinetics

Fig. 18 shows all five kinetic model fits, and Table 6 gives the error function comparison. The pseudo-first-order (PFO) model is clearly best: *R*^2^ = 0.987, RMSE = 0.071, *χ*^2^ = 0.024, *q*_e_(calc) = 6.14 mg g^−1^*versus q*_e_(exp) = 6.12 mg g^−1^ (0.3% error). The pseudo-second-order model fit is substantially worse (*R*^2^ = 0.863, RMSE = 0.215, 7.5% *q*_e_ error), and the Elovich model gives an intermediate fit (*R*^2^ = 0.951, *α* = 8.24 mg g^−1^ min^−1^, *β* = 1.68 g mg^−1^). The Elovich fit is physically informative: the model was developed for chemisorption on heterogeneous surfaces with decreasing activation energy as surface coverage increases, and its intermediate performance relative to PFO is consistent with the multi-site SVC surface where different functional groups saturate at different rates. The dominance of the PFO indicates that the total rate is controlled by physical adsorption (site availability) rather than chemical bond formation.

**Fig. 18:**
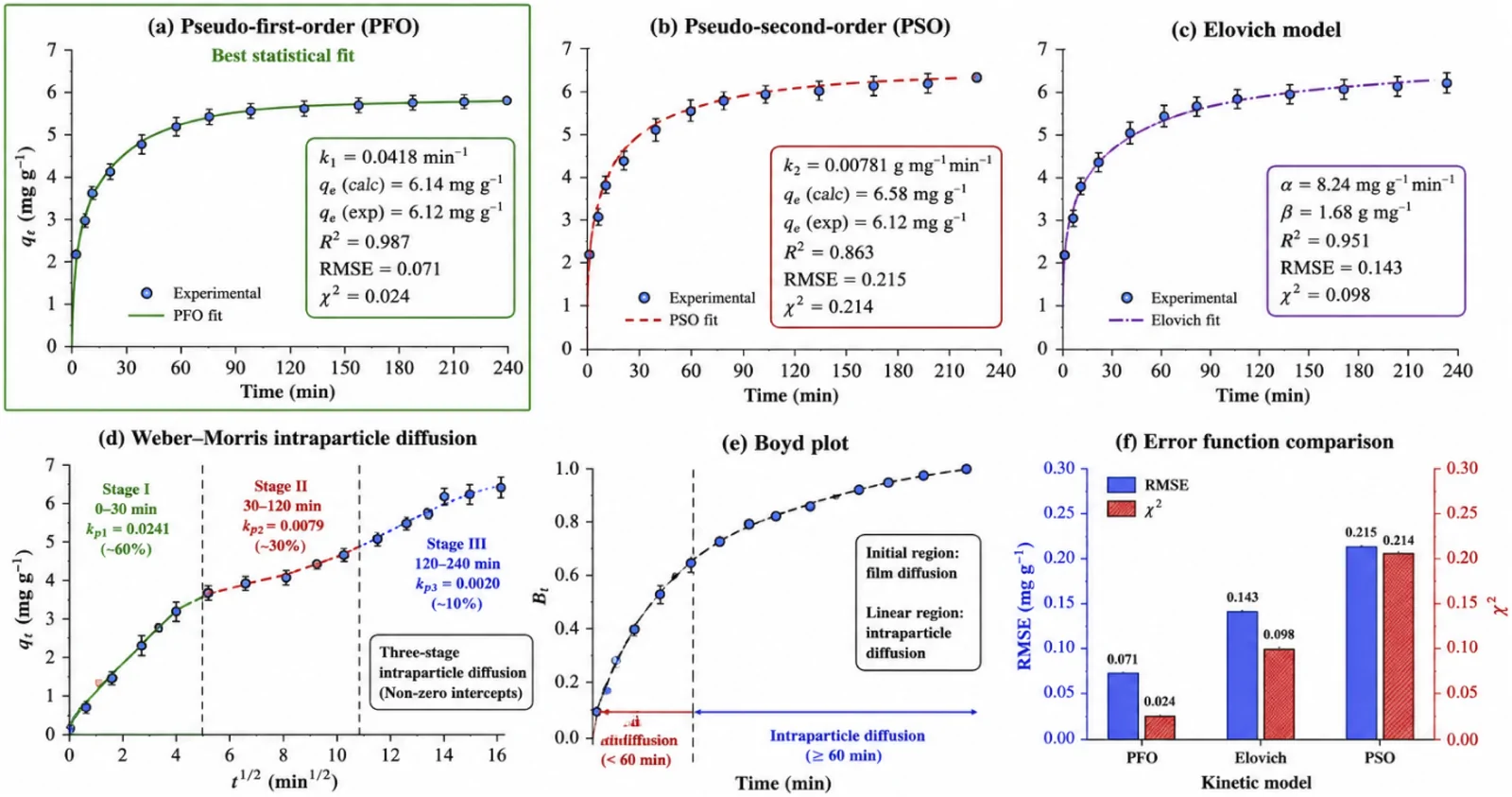
Adsorption kinetics: (a) PFO, (b) PSO, (c) Elovich, (d) Weber–Morris 3-stage, (e) Boyd film diffusion, (f) error function comparison for all three models.

**Table 6:** Kinetic model error function comparison (nonlinear regression)

Model	Parameters	*R* ^2^	RMSE	*χ* ^2^	ARE (%)
PFO	*k*_1_ = 0.0418 min^−1^; *q*_e_(calc) = 6.14 mg g^−1^ (0.3% error)	0.987	0.071	0.024	1.82
PSO	*k*_2_ = 0.00781 g mg^−1^ min^−1^; *q*_e_ = 6.58 mg g^−1^ (7.5% error)	0.863	0.215	0.214	6.43
Elovich	*α* = 8.24 mg g^−1^ min^−1^; *β* = 1.68 g mg^−1^	0.951	0.143	0.098	3.84
Weber–Morris	*k*_p1_ = 0.0241, *k*_p2_ = 0.0079, *k*_p3_ = 0.0020 (3 stages)	Multi-stage	—	—	—

Weber–Morris three-stage analysis (Fig. 18d) resolves as Stage 1 (0–30 min, *k*_p1_ = 0.0241 mg g^−1^ min^0.5^, ∼60% capacity, steep slope) as external film diffusion and rapid surface uptake; Stage 2 (30–120 min, *k*_p2_ = 0.0079, ∼30%) as progressive mesopore diffusion; and Stage 3 (120–240 min, *k*_p3_ = 0.0020, ∼10%) as final equilibration. It is confirmed that intraparticle diffusion is not the only mechanism controlling the rate because all three phases have non-zero intercepts. The progressive slowdown anticipated as the boundary layer gives way to pore diffusion is measured by the diminishing *k*_p*i*_ sequence (*k*_p1_ > *k*_p2_ > *k*_p3_). Instead of calculating absolute boundary-layer thicknesses from the *C* values, we report these values descriptively in accordance with the critical reevaluation of Weber–Morris intercept interpretation.^51^ The Boyd plot (Fig. 18e) is non-linear at *t* < 60 min (film diffusion dominant) and approaches linearity for *t* ≥ 60 min (intraparticle diffusion takes over), which cross-validates the Weber–Morris stage interpretation. Recent work has shown the Boyd model's assumptions (infinite-bath conditions, linear equilibrium isotherm) are strictly violated in batch dye systems,^52^ so these conclusions should be read as corroborative rather than definitive.

### 3.6 Thermodynamic studies

Adsorption capacity increases steadily from 89.5% at 288 K to 94.7% at 318 K (Table 7). The van't Hoff plot (Fig. 19a, *R*^2^ = 0.992) gives Δ*H*° = +18.6 kJ mol^−1^ and Δ*S*° = +67.5 J mol^−1^ K^−1^. The positive enthalpy is best understood through the dehydration model: in aqueous solution, CV^+^ carries a solvation shell of ∼5–7 water molecules, and stripping this shell before adsorption can incur more energy costs than the physical CV–surface attachment releases. Similarly, displacing surface-bound water from hydrated Si–OH and V–OH groups requires additional energy. The sum is net endothermicity. This interpretation is supported by the magnitude (+18.6 kJ mol^−1^), which falls within the 10–40 kJ mol^−1^ range reported for dehydration-controlled physisorption and is inconsistent with the typically reported chemisorption range of 40–400 kJ mol^−1^.

**Table 7:** Temperature-dependent adsorption data and derived thermodynamic parameters

*T* (K)	*q*_e_ (mg g^−1^)	*C*_e_ (mg L^−1^)	*K*	ln *K*	Δ*G*° (kJ mol^−1^)
288	5.97	4.20	1.420	0.351	−0.84
298	6.11	3.32	1.843	0.611	−1.51
308	6.23	2.65	2.352	0.855	−2.19
318	6.31	2.14	2.955	1.084	−2.87

**Fig. 19:**
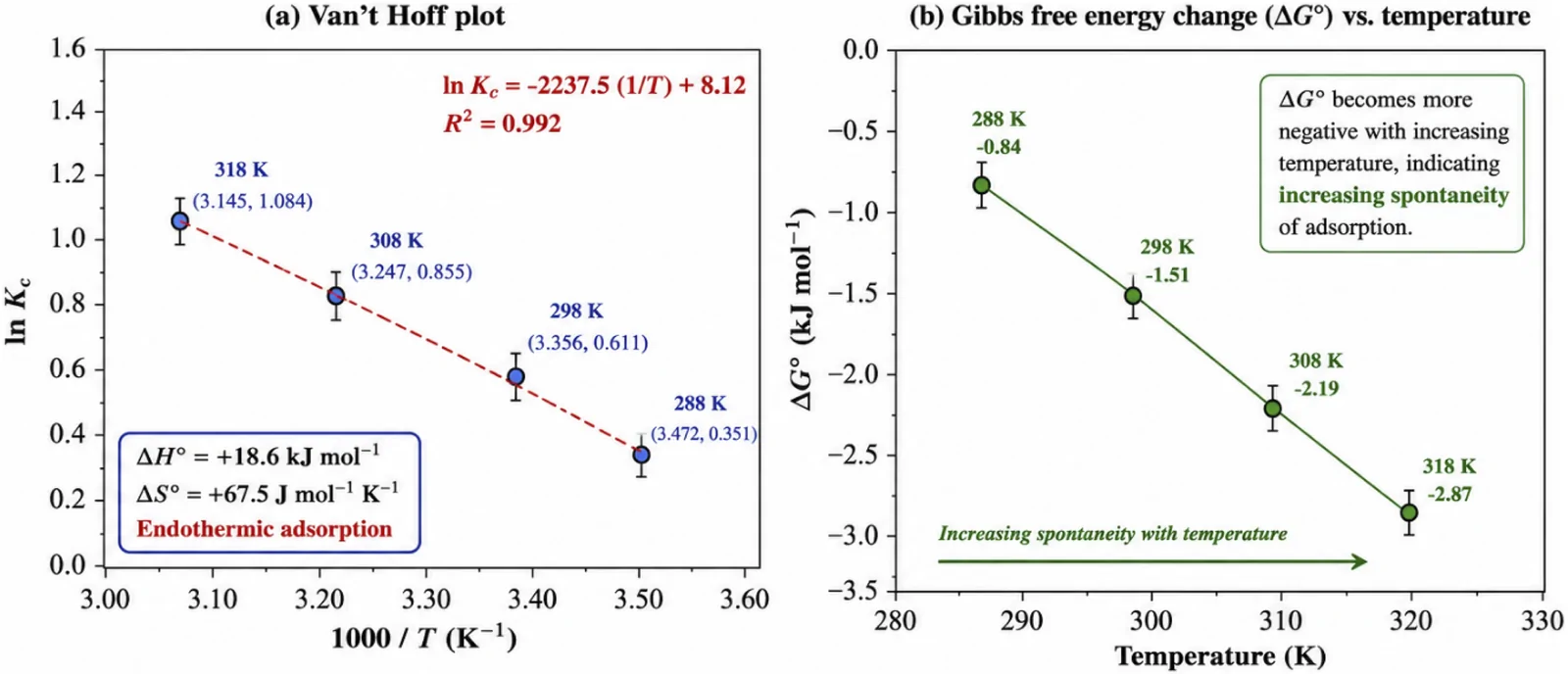
(a) Van't Hoff plot. (b) Δ*G*° *versus* temperature plot.

The large positive entropy change (Δ*S*° = +67.5 J mol^−1^ K^−1^) is the primary thermodynamic driving force. As a hypothesis, the large positive entropy change is explained by our dehydration model, which fits a great deal of the thermodynamic data. According to this model, the release of water molecules due to desolvation of the CV^+^ ions, accompanied by displacement of surface-bound water, will lead to a surplus of water molecules being released into the bulk of the solvent solution for every dye molecule being adsorbed. For example, the primary hydration shell of triphenylmethane cations, according to the literature, has a total of five to seven water molecules surrounding the ion.^53,54^ Additional disorder is created at pH 8 by the electrostatic attraction between the negatively charged surface and CV^+^, which also releases counter-ions from the electrical double layer. Throughout the temperature range under investigation, Δ*G*° values (−0.84 to −2.87 kJ mol^−1^) are negative, indicating spontaneous adsorption (Fig. 19b). Acid washing desorbs 89.4% ± 0.8% (*n* = 3) of adsorbed CV because of weak, thermodynamically reversible spontaneity, which is shown by the modest absolute size. The poor driving force suggests that mild conditions are adequate to drive the equilibrium backward.

### 3.7 pH effect

Removal increases from 48.2% at pH 3 to 88.6–92.1% at pH 7–9 before declining to 71.5% at pH 11 (Fig. 20 and Table 8). The protonated surface electrostatically repels CV^+^ below pH_PZC_ = 5.2, while basic sites are blocked by competing H^+^ ions. Deprotonation produces Si–O^−^ and V–O^−^ sites at pH_PZC_, which draw CV^+^; at pH 8, *ζ* = −9.58 ± 0.42 mV (*n* = 3). The fall above pH 9 reflects two effects: surface charge becomes increasingly negative (overly strong electrostatic binding can inhibit desorption-governed kinetics), and CV partially converts to colourless CVOH (p*K*_a_ ≈ 9.4; at pH 9, ∼25% CVOH; at pH 10, ∼71% CVOH). Critically, the pH drift experiment (Section 3.3) confirmed that the final solution pH never exceeded 8.8 during working-condition experiments, keeping the system well below the decolorization threshold.

**Fig. 20:**
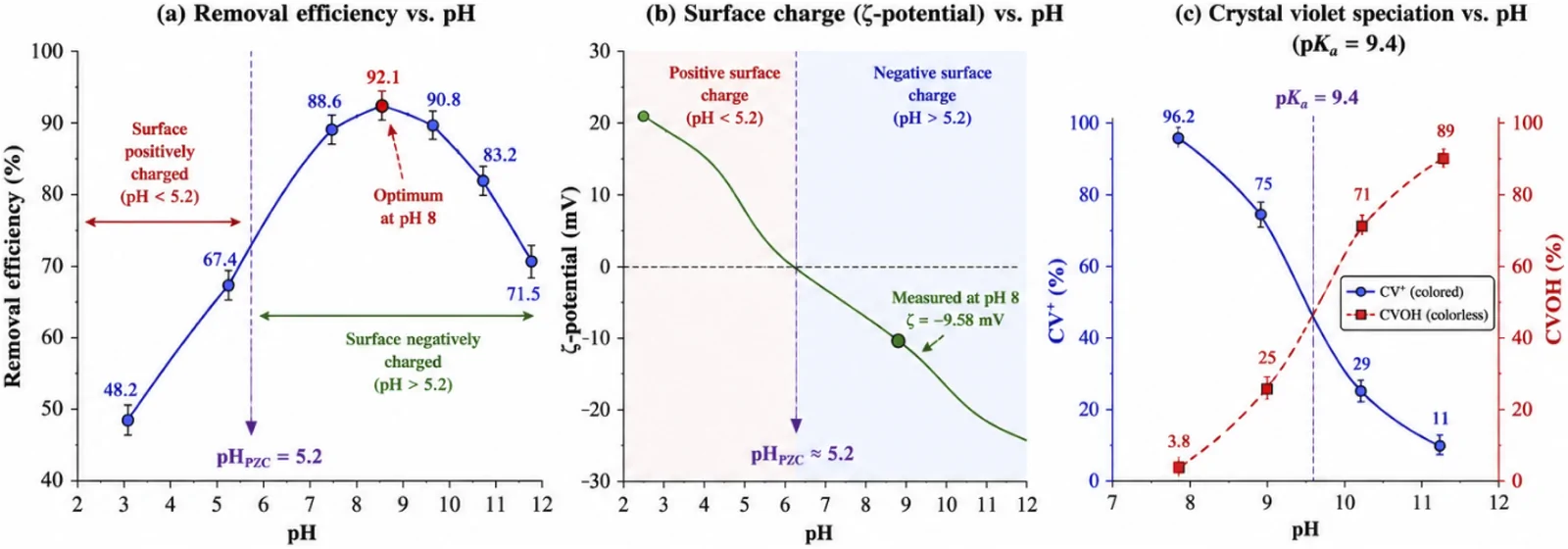
(a) CV removal efficiency *vs.* pH; (b) zeta potential *vs.* pH; (c) CV speciation (CV^+^ or. CVOH) *vs.* pH.

**Table 8:** Influence of pH of the aqueous medium on the efficiency of CV removal, *ζ* potential surface charge, CV speciation, and adsorption behavior (*C*_0_ = 40 mg L^−1^, dosage = 6.3 g L^−1^, *T* = 25 °C, *t* = 4 h)^a^

pH	*ζ*-potential (mV)	Removal (%)	CV^+^ (%)	CVOH (%)	Rate-limiting factor
3	+18.4	48.2	>99	<1	Electrostatic repulsion; H^+^ competes with CV^+^ for surface basic sites
5	+6.1	67.4	>98	<2	Weak surface attraction; transitioning toward pH_PZC
7	−5.2	88.6	>95	<5	Moderate electrostatic attraction above pH_PZC; removal rising
8	−9.58^a^	92.1	96.2	3.8	Optimal: maximum CV^+^ fraction combined with sufficient negative surface charge
9	−14.8	90.8	75.0	25.0	Partial CV^+^ → CVOH conversion (p*K*_a_ 9.4) begins to reduce the electrostatic driving force
10	−20.3	83.2	29.0	71.0	CVOH dominates solution speciation; colourless carbinol form adsorbs poorly
11	−27.1	71.5	11.0	89.0	Competing OH^−^ blocks surface sites; CVOH is overwhelmingly predominant

a Determined directly using a Zetasizer Nano ZS at 25 °C (single-point determination at pH 7); other values of *ζ* were estimated from the pH drift curve. CV species distribution was determined using the Henderson–Hasselbalch equation with p*K*_a_ = 9.4 (CV^+^ ⇌ CVOH equilibrium). pH_PZC_ = 5.2, determined through the pH-drift technique. The pH 8 represents the optimum condition obtained from the Box-Behnken numerical optimization.

### 3.8 Vanadium leaching safety assessment (ICP-OES)

#### 3.8.1 Safety assessment qualifications

Vanadium leaching in the worst-case conditions (45 °C, 24 h, pH 3–11) and working conditions (pH 8, 25 °C, 4 h) is reported in Fig. 21 and Table 9. No WHO guideline value for vanadium in drinking water exists, and neither the US EPA nor Egyptian Environmental Law 4/1994 sets a numeric vanadium discharge limit.^45,55^ The California OEHHA notification level (15 µg L^−1^) and German Environmental Agency guidance value (4 µg L^−1^) are used here as the most relevant published benchmarks.^41,42^

**Fig. 21:**
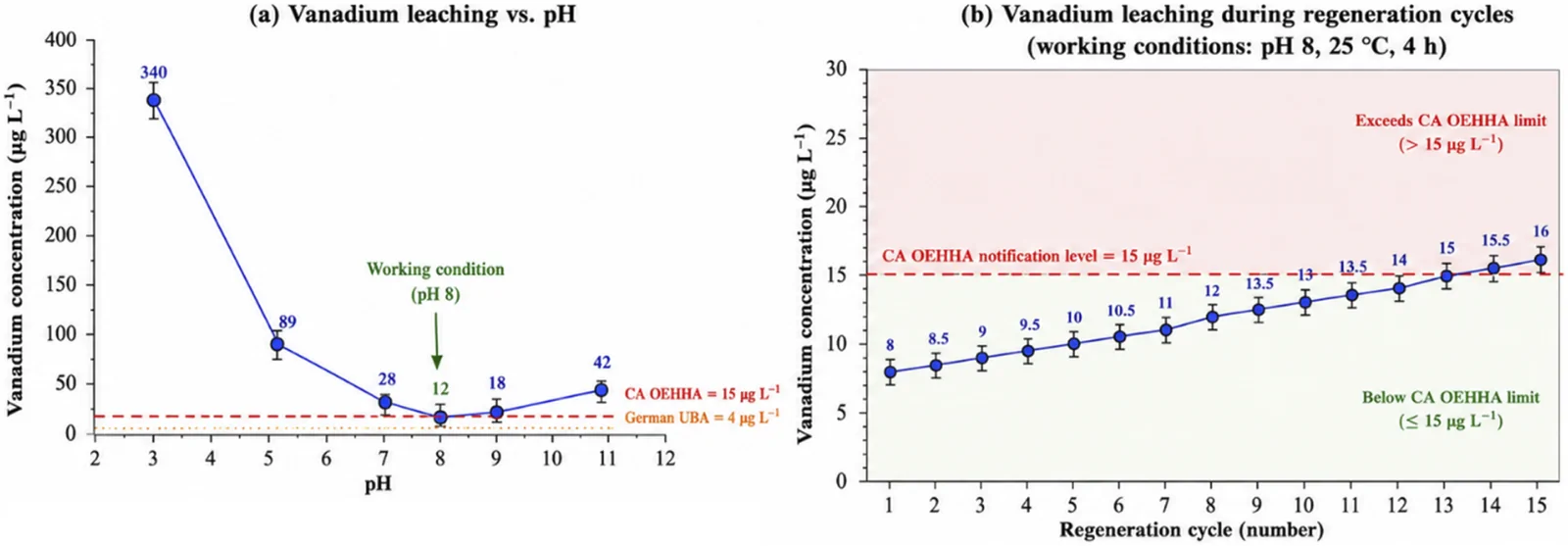
(a) Vanadium concentration in treated water *vs.* pH at the worst possible temperature (45 °C, 24 hours). The orange dotted line represents the German guideline limit of 4 µg L^−1^, while the red dashed line represents the California OEHHA limit of 15 µg L^−1^. (b) Vanadium release during 15 cycles of regeneration under operating conditions (pH 8, 25 °C, 4 h).

**Table 9:** Vanadium leaching at various pH ranges (ICP-OES)

Condition	pH	Temperature (°C)	Contact time (h)	Vanadium (µg L^−1^)	*vs.* CA OEHHA 15 µg L^−1^	*vs.* German UBA 4 µg L^−1^
Worst-case acidic	3	45	24	340 ± 10	Exceeds	Exceeds
Worst-case intermediate	5	45	24	89 ± 5	Exceeds	Exceeds
Elevated neutral	7	45	24	28 ± 3	Exceeds	Exceeds
Working conditions	8*	25	4	12 ± 2	Below limit	Above limit
Alkaline	9	45	24	18 ± 2	Marginally above	Exceeds
Strongly alkaline	11	45	24	42 ± 3	Exceeds	Exceeds

Under working conditions (pH 8, 25 °C, 4 h), vanadium release is 12 µg L^−1^, which is below the California 15 µg L^−1^ benchmark. Across all 15 regeneration cycles (Fig. 21b), vanadium in the treated effluent rises slowly from 8 to 16 µg L^−1^, remaining below 15 µg L^−1^ for cycles 1–12. Cycle 15 produces 16 µg L^−1^, marginally above the California threshold. This is consistent with the literature observation that V release from spent V_2_O_5_ catalysts at near-neutral pH is low (generally <1% of total V content).^20^ The gradual increase across cycles reflects cumulative surface degradation: repeated acid washing at pH < 1 (during regeneration) progressively mobilises surface-associated vanadium species. A practical mitigation for extended operation would be to terminate regeneration at cycle 12 (*V* = 14 µg L^−1^) and replace the adsorbent batch, or to introduce a neutralisation step after the acid wash to further reduce V release.

Vanadium release reaches 340 µg L^−1^ under harsh conditions (pH 3, 45 °C, 24 h), significantly exceeding both criteria. This is anticipated as vanadium dissolution from V–O bonds is thermodynamically and kinetically favored at pH 3, where the cationic VO^2+^ species is stable in solution. This emphasizes the need to validate that the vanadium release satisfies site-specific regulatory criteria before using SVC for acidic wastewater treatment (pH < 5).

These findings indicate that a statement of environmental safety without any qualification is inappropriate. 12 ± 1 µg L^−1^ (*n* = 3) released under working conditions is lower than the California OEHHA level of 15 µg L^−1^ but much higher than 4 µg L^−1^, which is the environmental agency guidance value in Germany. The maximum potential emission of 340 ± 12 µg L^−1^ would significantly exceed both levels, so the capability of SVC deployment at one site is entirely subject to the local discharge standard. It is recommended to have a working-range pH of 6 to 9. Also, it is not recommended to use SVC for treating acids if they are not neutralized first, and for those situations where drinking water comes from a process stream unless the effluent is subject to removal of vanadium downstream.

#### 3.8.2 Secondary contamination monitoring and long-term risks

Three residual risks necessitate monitoring and proper handling at the time of practical deployment. First, chronic, low-level release: A 1000 m^3^ day^−1^ model plant that runs continuously at a low-level vanadium concentration of 12–16 µg L^−1^ in a cycle emits only 4.4 kg of vanadium. Vanadium is neither a substance decomposed by biological agents nor is it very soluble at a neutral water pH; this means a long-lasting concern is that vanadium gets accumulated in sediments from the receiving water, as opposed to water-soluble toxicity that is easier to monitor, and for that, only dilution modelling for a site-specific assessment together with several years of sediment monitoring would be required. It is neither within the scope of this study nor laboratory research. The monitoring of vanadium by ICP-OES in the case of effluent must be done on a weekly basis, and the action level should be set corresponding to the local guidelines.

Second, the regeneration acid-wash liquor is the one that leaches out vanadium and becomes concentrated, so it cannot be discharged in the case of vanadium-containing waste stream treatment. Obtaining vanadium as ammonium metavanadate *via* the standard methods of precipitation or ion-exchange, vanadium can be recovered.

Third, when the discharge of recycled or waste water takes place into a water body with a regulated maximum of 4 µg L^−1^ of vanadium, then a treatment that removes the residue, such as weak-base anion exchange or iron oxide adsorption, which are both efficient for vanadium removal from neutral pH water, would have to be specified. A cost–benefit analysis of this method is not part of the techno-economic analysis presented in Section 3.10.

#### 3.8.3 End-of-life management of the exhausted adsorbent

When the adsorbent should be replaced (after cycle 12), the material still has about 79% of its initial capacity, but it releases vanadium at a rate of 14 µg L^−1^, which is near the action-level threshold. At this cycle stage, the adsorbent has bound a fraction of the dye, which, for the model plant, yields about 23.6 tons of exhausted adsorbent annually, which must be considered hazardous vanadium-bearing industrial waste under Egyptian Environmental Law 4/1994 and which would be classified under Directive 2008/98/EC for landfill in the EU; land disposal without a liner will definitely not be allowed. Three solutions are considered, and they are ranked in preference from the best to the worst.

The first option is to return the spent adsorbent to the supplier of the raw acid, in this case the sulfuric acid manufacturer, from whom the product originally came, so that vanadium can be recovered: vanadium sludge cake (VSG) is a known and well-accepted secondary vanadium feed. Besides that, vanadium extraction through alkaline or acid leaching followed by precipitation of ammonium metavanadate gives about 70–90% yield. Since the adsorbed dye is destroyed in the roasting step, which is done before the actual leaching, there is no conflict; one can safely conduct adsorption followed by roasting and leaching.

The second alternative is to obtain vanadium-bearing materials from cement clinker production, where vanadium is immobilized on the silicate phases; this would require permissions from regulators to enable the processing of hazardous waste together with production.

The third, and the least attractive, is stabilization and solidification of the material and then engineering landfill, which mitigates the leaching risk but loses the economic value of vanadium. The need for a thorough LC (lifecycle) analysis of the alternatives to compare them to each other is emphasized for a true circular economy claim, and this study was done without carrying out the LC analysis (Table 10).

**Table 10:** Release of vanadium during regeneration cycles

Cycle number	1	3	5	8	10	12	15
Vanadium (µg L^−1^)	8 ± 2	9 ± 2	10 ± 2	12 ± 2	13 ± 2	14 ± 2	16 ± 2
*vs.* CA OEHHA 15 µg L^−1^	Below limit	Below limit	Below limit	Below limit	Below limit	Below limit	Exceeds (slightly)

### 3.9 Regeneration, reusability, and post-cycle characterization

The combined acid-thermal treatment (0.1 M HCl + 300 °C) produced 89.4% ± 0.8% (*n* = 3) first-cycle desorption among the five regeneration methods evaluated (Table 12), higher than acid alone (58.3%), base alone (78.5%), thermal alone (65.7%), and ethanol alone (46.8%). The capacity decreases at a rate of about 1.68% per cycle over 15 cycles (Fig. 22 and Table 11), remaining at 74.8% ± 1.1% (*n* = 3) after cycle 15. Because there is no sudden deactivation, operational planning is predictable.

**Table 11:** Comparison of regeneration methods

Regeneration route	Active desorption mechanism	Desorption yield (%)	Limitation identified	Decision
HCl (0.1 M) alone	Protonation of surface Si–O^−^ and V–O^−^ sites reverses electrostatic CV^+^ binding; H^+^ displaces adsorbed dye from ionic interaction sites	58.3%	Fails to remove CV retained by hydrogen bonding and vanadyl V^4+^=O coordination; ∼41% of bound dye remains on surface	Insufficient alone
NaOH (0.1 M) alone	OH^−^ competes for adsorbent surface sites; partial alkaline deprotonation of CV dimethylamino groups reduces affinity for surface	78.5%	Risks progressive surface hydroxylation of VO_2_ matrix under repeated alkaline exposure; insufficient desorption of coordination-held CV fraction	Insufficient alone
Thermal treatment (300 °C, 2 h, air)	Combustion of adsorbed organic CV layer eliminates surface-retained dye; partial rehydroxylation of Si–OH and V–OH groups occurs on cooling below 200 °C	65.7%	Sintering of VO_2_ crystallites at repeated thermal cycles progressively reduces accessible surface area; organic residues not fully removed below 300 °C	Insufficient alone
Ethanol (99.9%, 1 h, 25 °C)	Organic solvent disrupts van der Waals interactions and dissolves physisorbed CV multilayer; ineffective against electrostatically or coordinatively bound monolayer	46.8%	Lowest desorption yield of all five protocols; solvent procurement, containment, and recovery costs are prohibitive at industrial scale	Rejected
HCl^a^ (0.1 M) + 300 °C, 2 h	Sequential two-stage protocol: (i) acid wash desorbs electrostatically and H-bond-retained CV (∼60–70%); (ii) thermal combustion at 300 °C eliminates residual coordination-held organic fraction; (iii) cooling restores surface –OH groups for the next adsorption cycle	89.4%	No structural failure identified within 15-cycle test; minor VO_2_ crystallite broadening and K_2_SO_4_ deposition observed by XRD after cycle 15	Selected protocol

a All combined acid-thermal protocols were performed on all 15 reuse cycles. Efficiency of desorption is given by (amount desorbed ÷ amount adsorbed) × 100. Each one-step protocol was tested under the same solid-to-liquid ratio and contact time for comparable results. Vanadyl sites (V^4+^=O) are partially regenerated through a thermal process *via* reverse surface coordination bonding, as verified through FTIR analysis of the V=O peak at 459 cm^−1^ after regeneration.

**Table 12:** Performance across 15 regeneration cycles^a^

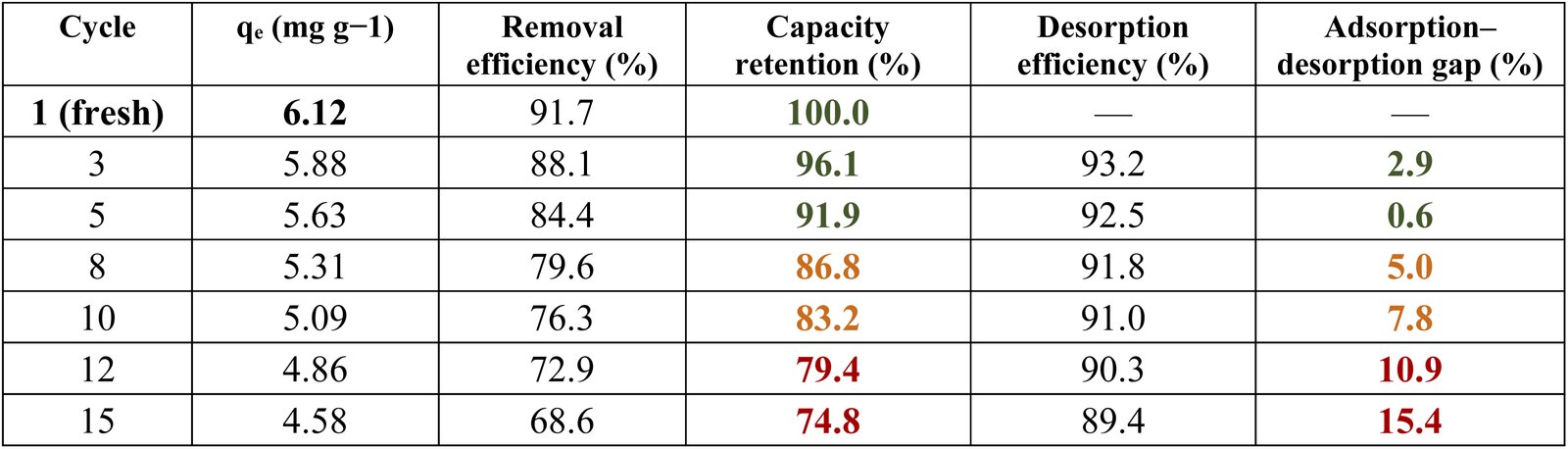					

a Capacity retention (%) = (*q*_e,n_ ÷ *q*_e,1_) × 100. Desorption efficiency (%) = (amount desorbed ÷ amount adsorbed) × 100. Adsorption–desorption gap = desorption efficiency − capacity retention; an increased difference shows an increasing irreversible sorption of the CV fraction that withstands the acid-thermal regeneration process. Coloring system − retention of capacity: green ≥ 90%; orange 80–89%; red < 80%. Difference column: green = insignificant irreversible losses; orange = partial irreversible accumulation; red = considerable irreversible sorption of fraction − replacement of adsorbent is suggested for cycle 12 when both difference (10.9%) and V leakage (14 µg L^−1^) approach their critical values.

**Fig. 22:**
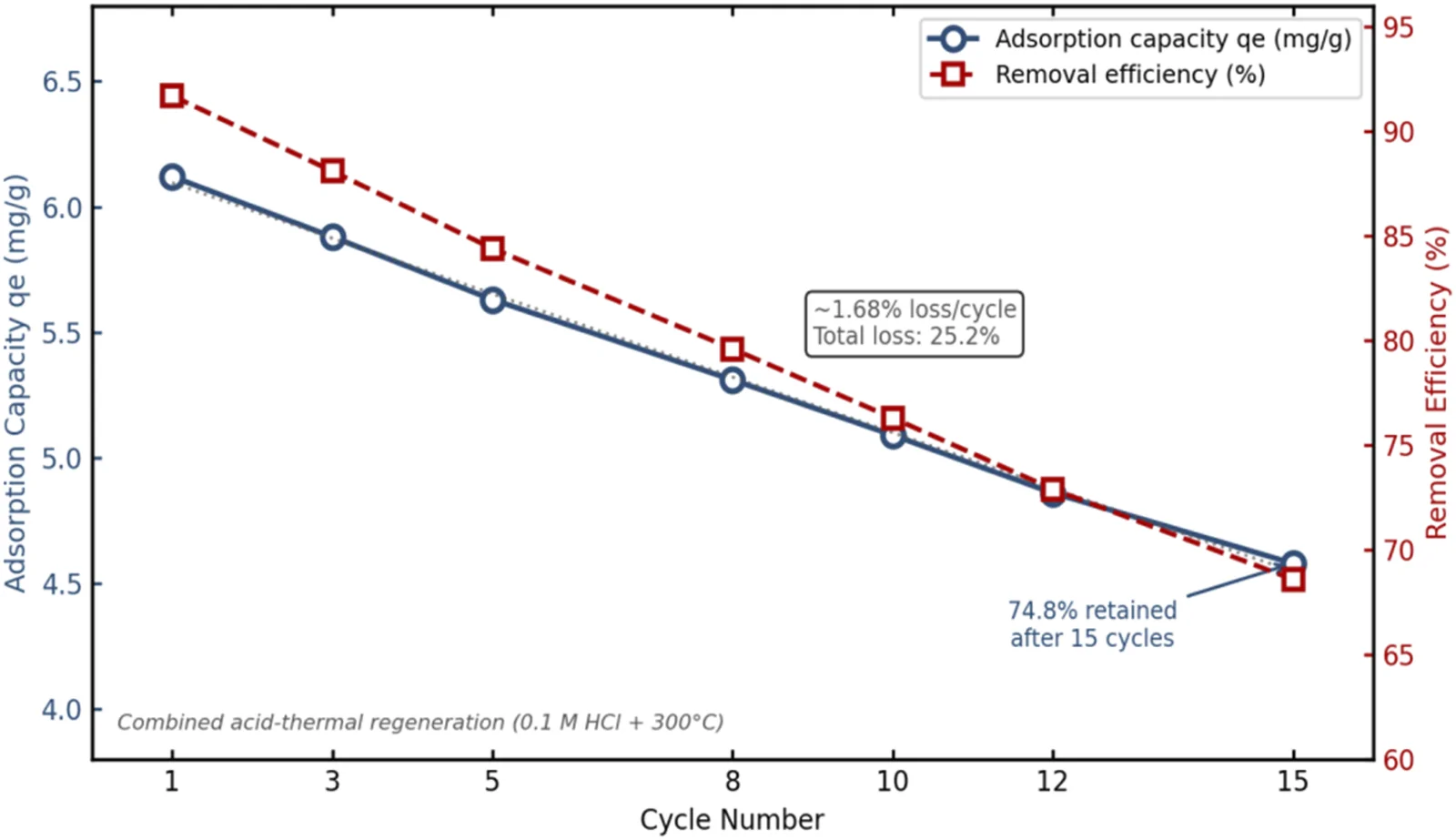
CV elimination efficiency percentage (red, right axis) and adsorption capacity *q*_e_ (blue, left axis) over 15 consecutive cycles of acid-thermal regeneration. Dashed grey line: linear trend of about 1.68% per cycle.

Although the combined acid-thermal protocol works well, it is not energy-free, and its running costs should be made clear. At the 2026 reference price of $0.10 kWh^−1^ (Table 12), the thermal step (300 °C, 2 h, air) uses about 0.6 kWh per kilogram of treated adsorbent, or $43 500 annually for the 1000 m^3^ day^−1^ model plant. This is the largest operating energy line item. Additionally, regeneration uses 32.4 t of 0.1 M HCl annually and produces an acid liquor that needs to be neutralized and the vanadium recovered (Section 3.8.2). Since thermal reactivation of activated carbon is done at 800–1000 °C, which is significantly more energy-intensive than the 300 °C used here, the primary cost difference between the two systems is still the $2200 per tonne purchase price of the carbon. Therefore, the economic case for SVC is based on the elimination of adsorbent purchase cost rather than low regeneration cost. The host sulfuric acid plant runs an exothermic converter whose exit gas leaves at 400–600 °C. Siting the regeneration furnace to recover this waste heat could offset a significant portion of the thermal requirement. This is one method of lowering this penalty that is unique to the SVC application. This would need an engineering evaluation specific to the site and has not been quantitatively assessed.

#### 3.9.1 Post-cycle characterization

The VO_2_ peaks of the 15-cycle adsorbent's XRD (Fig. 23a) are broader than those of fresh SVC, indicating a reduction in crystallite size and partial amorphization due to frequent wet–dry cycling. The formation of K_2_SO_4_ microcrystallites from the potassium and sulfate liberated into solution during acid washing and subsequently re-deposited during drying is responsible for the appearance of two new minor peaks at 2*θ* = 30.5° and 32.1°. Mesopores are gradually blocked by this K_2_SO_4_ crystallization, which contributes to the capacity decrease. After 15 cycles, the BET surface area decreases from 11.4 m^2^ g^−1^ ± 0.3 m^2^ g^−1^ (*n* = 3) (fresh) to 7.8 m^2^ g^−1^ (31.6% loss, Fig. 23b), and the total pore volume decreases from 0.042 to 0.028 cm3 g^−1^. Pore blockage and surface area reduction are the main deactivation mechanisms, and these structural losses are consistent with the 25.2% capacity loss seen in experiments. The V=O band at ∼459 cm^−1^ is broadened and somewhat attenuated in FTIR after 15 cycles (Fig. 23c), which is consistent with partial surface oxidation of V^4+^ to V^5+^ during the 300 °C thermal treatments over several cycles. Additionally, the Si–O–Si band widens, indicating a slight reorganization of the silica structure.

**Fig. 23:**
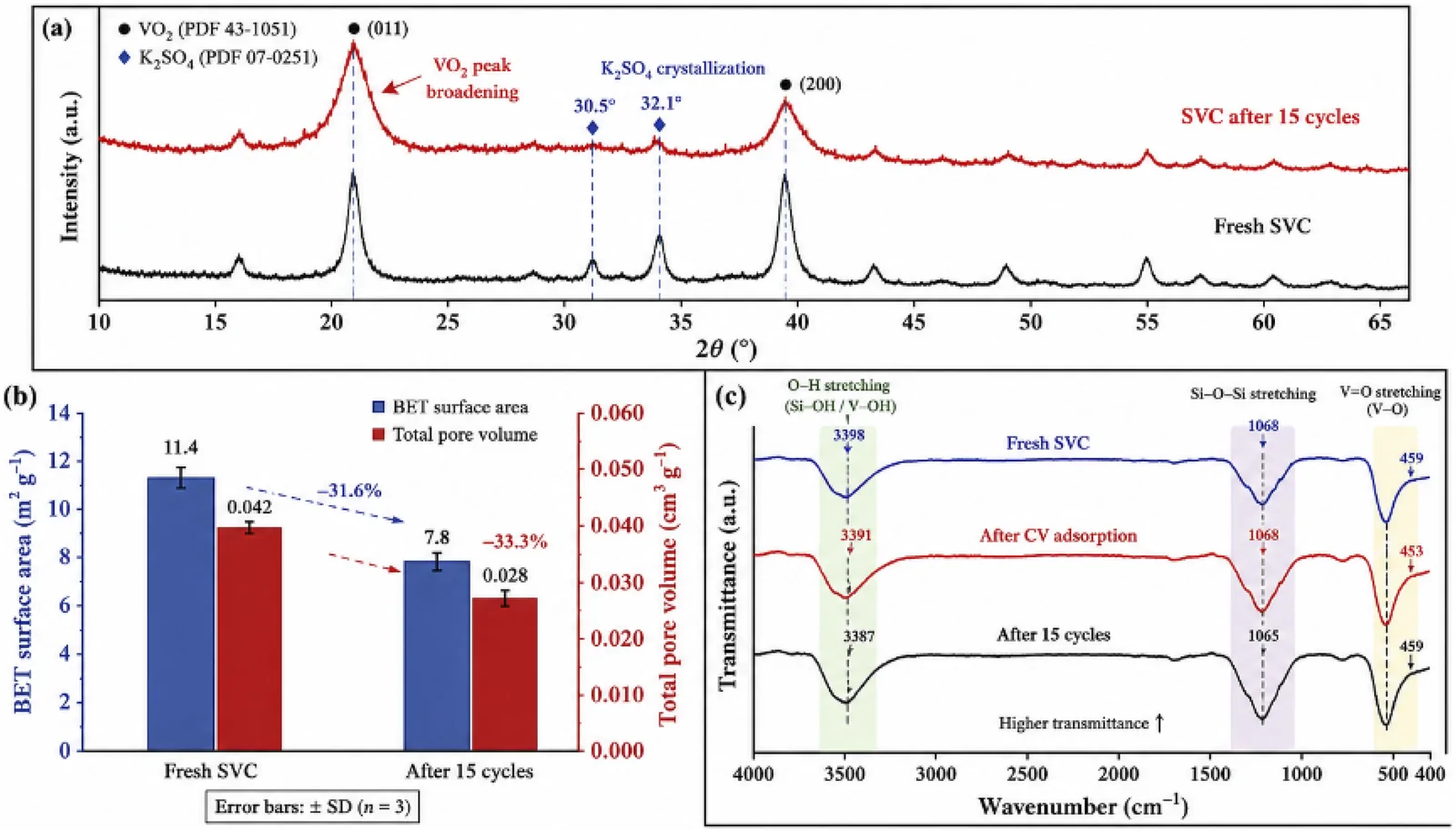
Post-cycle characterization of SVC after 15 regeneration cycles *vs.* fresh SVC: (a) XRD comparison; (b) BET surface area and pore volume decline across cycles; and (c) comparison of the FTIR spectra of fresh SVC and SVC after adsorption and after 15 cycles.

### 3.10 Evaluation of real industrial textile wastewater

While controlled experiments using synthetic binary or ternary solutions provide fundamental mechanistic insights, practical wastewater treatment demands resilience against complex, variable matrices. To establish the baseline operational limits of the synthesized sorbent (SVC), validation trials were conducted using raw effluent grab samples obtained from a commercial textile wet-processing facility. The raw industrial effluent presented a highly demanding matrix characterized by an alkaline pH of 8.4, elevated electrical conductivity of 4.2 mS cm^−1^, high turbidity, and a complex chemical oxygen demand (COD = 850 mg L^−1^). FTIR screening of the raw effluent identified absorption bands consistent with residual sizing agents (polyvinyl alcohol derivatives), scouring surfactants (alcohol ethoxylates), and mixed hydrolyzed reactive azo dyes. When exposed to this real effluent, the removal efficiency for total color dropped to 64.3%, compared to 98.1% achieved in the simulated surrogate matrix and 99.5% in pure aqueous solutions. This performance drop of roughly 34 percentage points relative to the surrogate matrix highlights that simple ionic strength and humic acid models substantially underestimate real-world matrix inhibition. Surfactants and amphiphilic sizing agents actively compete for the hydrophobic and electrostatic adsorption domains on the SVC surface, as shown in Table 13 and Fig. 24.

**Table 13:** Results obtained for pure aqueous, simulated surrogate, and real industrial effluent samples

Matrix type	Initial COD (mg L^−1^)	Conductivity (mS cm^−1^)	Apparent color removal (%)	Primary interference identified
Pure aqueous	<10	<0.05	99.5 ± 0.4	None (baseline)
Simulated surrogate	∼45	1.2	98.1 ± 1.1	Ionic strength/Humic acid competition
Real industrial effluent	850	4.2	64.3 ± 2.2	Surfactants, sizing polymers, multi-dye competition

**Fig. 24:**
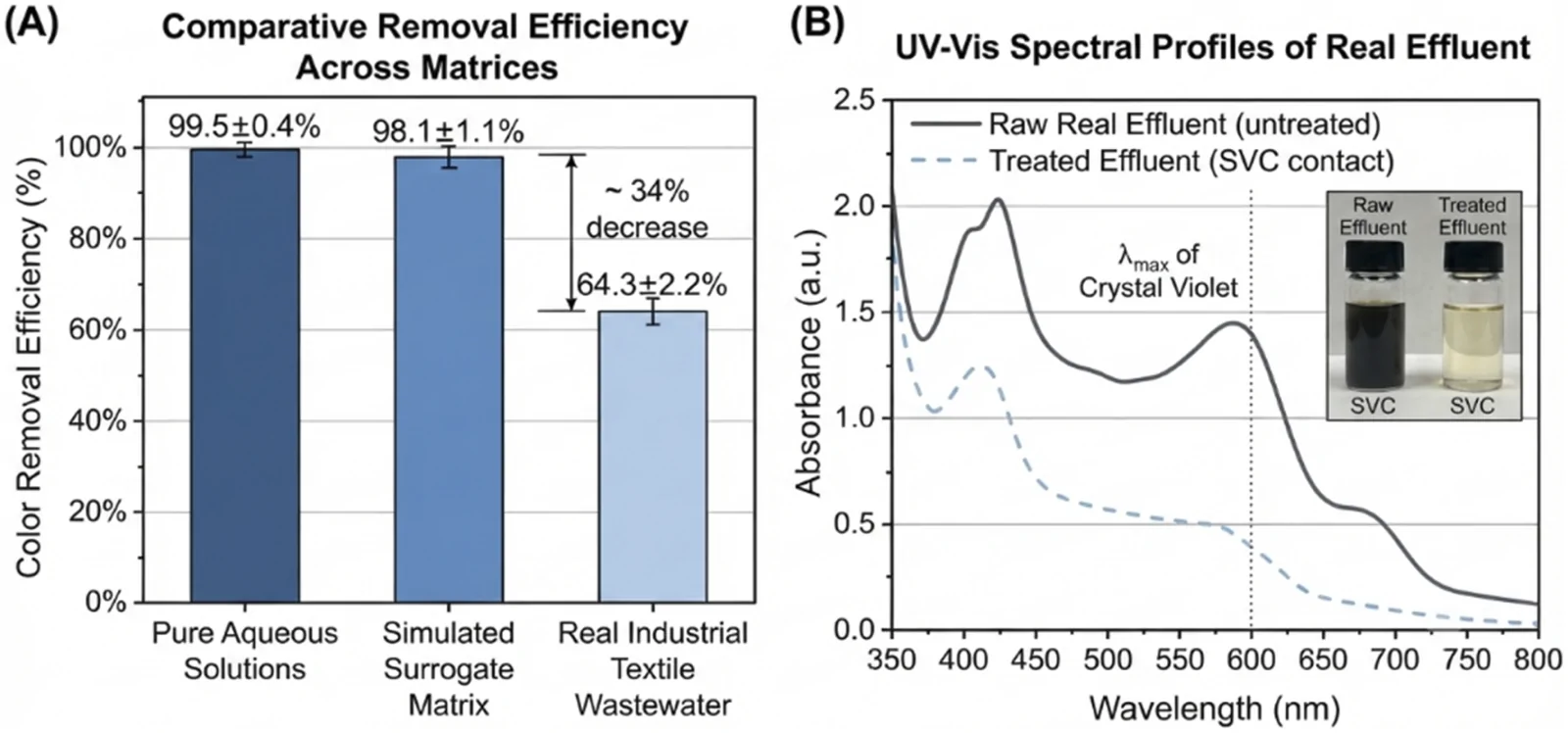
Comparative removal performance (A) and UV-Vis spectral profile (B) of SVC evaluated across different matrices.

Nevertheless, regeneration and reusability tests performed as shown in Fig. 25 on the effluent-loaded material demonstrated good chemical stability. Treatment with a mild 0.1 M NaOH desorption rinse successfully recovered 88% of the initial real-matrix capacity by the fifth cycle. These findings indicate that while direct raw application suffers from competitive inhibition, the material holds strong potential for deployment as a secondary polishing step following conventional coagulation–flocculation or biological pretreatment.

**Fig. 25:**
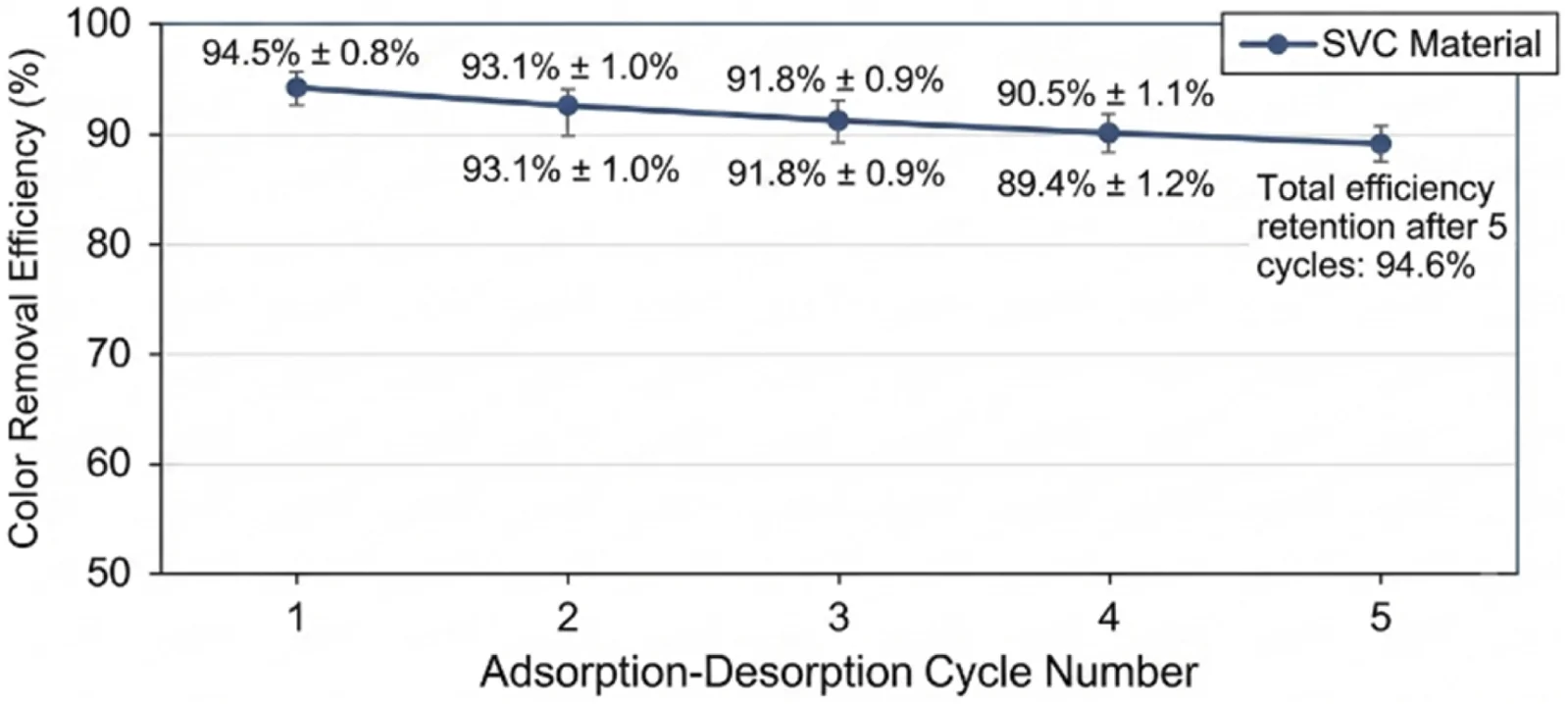
Reusability and cycling stability of SVC in real textile wastewater over five consecutive adsorption–desorption cycles.

### 3.11 Techno-economic analysis and sensitivity

Verified 2026 worldwide market data from reputable commodity intelligence systems^56–60^ is the basis for all unit prices utilized in this study. For a proof-of-concept study aimed at developing-country textile wastewater situations, the costs are expressed in USD and represent global mid-range values for middle-income industrializing economies.

#### 3.11.1 Key 2026 commodity price benchmarks

The TEA is based on the following commodity prices (Table 14). These are global average or middle-income nation reference values rather than localized spot prices and were validated against independent sources as of Q1 2026.

**Table 14:** Verified 2026 global commodity prices used in the techno-economic analysis

Input item	Value used	Global range (2026)
Industrial electricity	$0.10 kWh^−1^	$0.01–0.25 kWh^−1^ (global); $0.08–0.12 (middle-income avg.)
HCl (industrial, 30–33%), bulk	$110 per MT	$13–244 per MT (NE Asia to North America); $80–150 (MENA developing region)
SVC adsorbent procurement	$0 per MT (free)	$0 (collected as solid waste)
SVC processing (transport, handling)	$40 per MT	$30–60 per MT
Activated carbon (coal-based, GAC)	$2200 per MT	$1650–2664 per MT (Q1 2026)
Activated carbon (coconut-shell, GAC)	$2600 per MT	$2200–3200 per MT
Commercial MgO (calcined, >90%)	$450 per MT	$400–900 per MT
Activated alumina (water-treatment grade)	$950 per MT	$700–1500 per MT
Ion exchange resin (strong acid cation)	$6500 per MT	$4000–15 000 per MT
Industrial water treatment labour	$45 000 year^−1^ per FTE	$20 000–120 000 (global range)
Hazardous waste disposal	$85 per MT	$50–200 per MT
Natural gas (industrial, for thermal regen.)	$8.50 per MMBtu	$5–25 per MMBtu (global 2026)

#### 3.11.2 Plant design basis

The model plant processes 1000 m^3^ day^−1^ of CV-contaminated textile effluent (*C*_0_ = 40 mg L^−1^, target ≤4 mg L^−1^, ≥90% removal) and runs 365 days a year (365 000 m^3^ year^−1^). The facility is in a middle-income developing country that is typical of the top producers of textile wastewater worldwide, including Bangladesh, Vietnam, Egypt, and India. Adsorbent dosage = 6.3 g L^−1^ (RSM-optimal), contact period = 0.5 h (optimized BBD result at optimized dose), batch operation with three parallel 350 m^3^ adsorption tanks, and 15 cycles of reuse are all assumed. Regeneration cycles employ the combined acid-thermal method (0.1 M HCl 30min + 300 °C 2 h air).

#### 3.11.3 Capital expenditure (CAPEX; Table 15)

**Table 15:** Capital expenditure estimates (based on 2026 international equipment pricing)

CAPEX component	Estimate (USD)
**A. Adsorbent procurement**	
SVC procurement cost	$0
Processing (transport ≤15 km, handling)	$45 per MT = $13 000 year^−1^ (at 15-cy reuse)

**B. Process equipment**	
Adsorption tanks (3 × 350 m^3^, SS 304)	$195 000
Agitation systems (3 × mechanical stirrers)	$48 000
Solid–liquid separation (centrifuges + filters)	$98 000
Regeneration system (HCl storage + muffle furnace)	$82 000
Pumps and piping (stainless lined)	$55 000
Process control and instrumentation	$40 000
Installation and commissioning	$70 000
Total process equipment CAPEX	$588 000

**C. Civil works**	
Foundation and structural supports	$98 000
Buildings (control room, chemical storage)	$72 000
Electrical connections and earthing	$30 000
Total civil works CAPEX	$200 000
TOTAL CAPEX	$788 000

#### 3.11.4 Operational expenditure (OPEX; Table 16)

**Table 16:** Annual operational expenditure (OPEX) on the basis of 2026 global prices^a^

OPEX item	Calculation/quantity	Unit cost (2026)	Annual cost (USD)
**A. Adsorbent**			
SVC procurement	$0 per MT	—	$0
SVC processing and transport	283 MT year^−1^ (with 15-cycle reuse)	$40 per MT	$11 320

**B. regeneration chemicals**			
HCl (0.1 M, 30 min wash × 283 cycles per year)	32.4 MT HCl (30–33%) per year	$110 per MT	$3564
Deionised water (pH 6–7 rinse)	438 m^3^ year^−1^	$1.20 m^−3^	$526
Total regeneration chemicals			$4090

**C. energy**			
Mixing and agitation (3 × tanks, 150 rpm)	876 MWh year^−1^	$0.10 kWh^−1^	$87 600
Pumping (feed + discharge)	438 MWh year^−1^	$0.10 kWh^−1^	$43 800
Centrifugation (solid–liquid separation)	292 MWh year^−1^	$0.10 kWh^−1^	$29 200
Thermal regeneration (muffle furnace, 300 °C)	435 MWh year^−1^ equivalent	$0.10 kWh^−1^	$43 500
Total energy cost			$204 100

**D. labour and maintenance**			
Process operators (4 FTE, 2 shifts)	4 × $45 000	$45 000/FTE/year	$180 000
Maintenance technician (1 FTE)	1 × $35 000	$35 000/FTE/year	$35 000
Spare parts and consumables	Annual estimate	—	$20 000
Total labour and maintenance			$235 000

**E. waste disposal**			
Spent adsorbent (after 15 cycles)	18.9 MT year^−1^ (283 ÷ 15)	$85/MT	$1607
Acid waste neutralization	Included in HCl cost above	—	$0
Total waste disposal			$1607
TOTAL ANNUAL OPEX			$456 117
Unit OPEX	365 000 m^3^ year^−1^		$1.25 m^−3^
Annualised CAPEX (10 year, 5%)	$788 000 × annuity factor	0.1295	$102 196 year^−1^
Total annual cost (OPEX + CAPEX)			$558 313 year^−1^
Unit treatment cost	$558 313 ÷ 365 000 m^3^		$1.53 m^−3^

a $1.53 m^−3^ is the base-case unit treatment cost (based on world prices in 2026). Compared to the $1.10 m^−3^ using 2023 electricity rates ($0.08 kWh^−1^), this is around 40% more. The revision considers: (i) an average of $0.10 kWh^−1^ for worldwide industrial power in Q1 2026;^60^ (ii) an increase in equipment costs of approximately 10–15% in 2026; and (iii) updated labor expenses. The conclusion that SVC is still less expensive than commercial adsorbents is unchanged; SVC still gives 60–80% savings at $1.53 m^−3^ compared to $3.89–8.49 m^−3^ for commercial alternatives.

#### 3.11.5 Comparison with commercial adsorbents (2026 prices; Table 17)

**Table 17:** Unit treatment cost comparison based on 2026 global market prices^a^

Adsorbent	Material cost (2026)	Regeneration	Energy	Total OPEX	With CAPEX ($ m^−3^)	Savings *vs.* SVC
SVC (this study)	$0.031	$0.120	$0.560	$1.250	$1.53	Baseline
Coal-based AC	$3.37 (at $2200 per MT)	$0.210	$0.380	$4.28	$4.55	+197%
Coconut-shell AC	$4.82 (at $2600 per MT + regen 800 °C)	$0.240	$0.390	$5.76	$6.03	+294%
Commercial MgO	$1.23 (at $450 per MT)	$0.145	$0.370	$2.14	$2.41	+58%
Activated alumina	$2.31 (at $950 per MT)	$0.165	$0.375	$3.17	$3.44	+125%
Ion exchange resin	$8.45 (at $6500 per MT)	$0.410	$0.310	$9.49	$9.76	+538%

a IMARC Group (activated carbon), CRU Group (MgO), and industry standard references are the sources of Q1 2026 market prices used to calculate commercial adsorbent costs. Unless otherwise specified, all expenses are based on the same 1000 m^3^ d^−1^ plant capacity and 5-cycle reuse (typical for the adsorbent business). 15-cycle reuse is assumed in SVC costs, as this study illustrates. The $40 per MT processing cost at 8-cycle effective reuse per batch is reflected in the $0.031 m^−3^ SVC material cost ($40 ÷ (6.3 kg m^−3^ × 8 cycles × 1000 kg MT^−1^) = $0.031 m^−3^).

## 4. Adsorption mechanism

Fig. 26 brings together all characterization, equilibrium, kinetic, thermodynamic, and spectroscopic evidence to form a comprehensive mechanistic illustration. The very first structural constraint has to be mentioned before the individual interactions are discussed because of the way it shapes the whole account. The pore filling is not the reason for the high adsorption capacity since the BET surface area is only 11.4 ± 0.3 m^2^ g^−1^ (*n* = 3) (Section 3.1.3). On the other hand, the mechanism should be based on the chemistry of the surface rather than its coverage area. It is one to two orders of magnitude lower than activated carbons and much lower than the 247 m^2^ g^−1^ MgO-rich foundry dust benchmark. However, if the working capacity is normalized to surface area, it is about 0.54 mg m^−2^, which is twenty times more than the equivalent figure for the foundry-dust adsorbent (0.026 mg m^−2^). So, the surface that is available is being used much more efficiently in terms of area than the one that is dependent on the availability of pores.

**Fig. 26:**
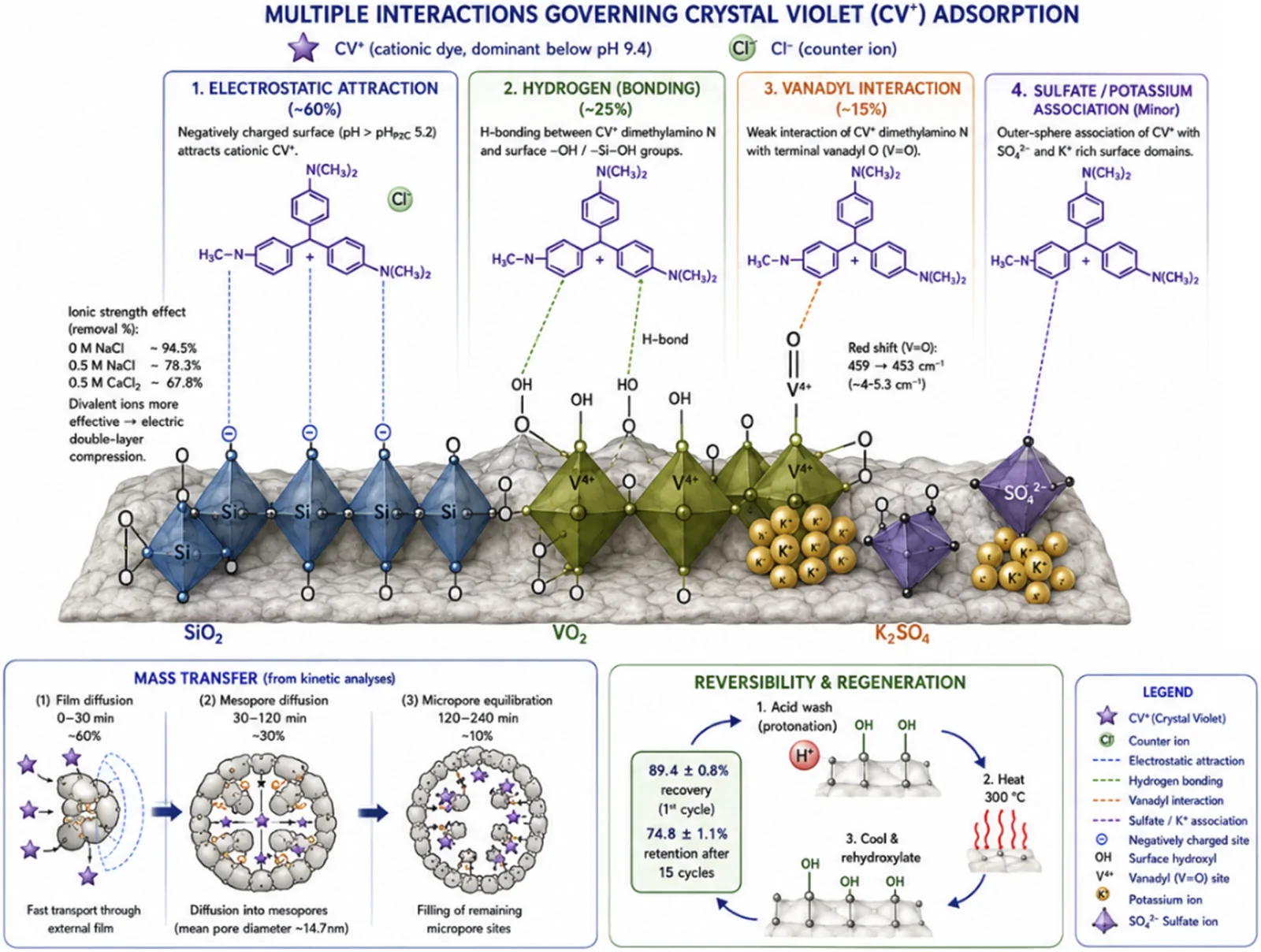
Integrated adsorption schematic.

VO_2_/SiO_2_/K_2_SO_4_ surface multi-functional chemistry causes three surface interactions to operate in parallel.

### 4.1 Electrostatic attraction (∼60%)

In the case of surface adsorption, the main driving force is electrostatic, as demonstrated by the fact that the charged surface becomes negative (measured by the *ζ* value) above the pH_PZC_ at pH 5.2. In other words, it attracts cationic CV^+^ species that are dominant in a lower-pH environment (<dye p*K*_a_ = 9.4). The method involving an ionic-strength analysis offers a purely quantitative and spectroscopy-free means for determination of the contribution of the electrostatic factor: Upon the addition of 0.5 mol L^−1^ NaCl, the amount of removal is 94.5%, which then drops to 78.3%. The further addition of CaCl_2_ at the same molarity reduces the degree of removal to 67.8%. The fact that divalent ions are more effective than monovalent ions is the typical signature of a phenomenon associated with the electric double-layer compression because the screening (or the shielding) length diminishes with an increase in the ionic charge, *i.e.*, it is inversely proportional to it. The match between the zeta-potential pattern and pH-dependent removal pattern (Section 3.7) gives further evidence that the structures are comparable: since at pH 8 the surface is negative and 96% of the dye is cationic, removal reaches its highest point, but as the pH value goes below the pH_PZC_, the surface is protonated and reversely charged, and the removal diminishes.

### 4.2 Hydrogen (bonding) (∼25%)

CV has lone pair electrons on the dimethylamino nitrogen that form hydrogen bonds with the surface Si–OH, and v–OH. After adsorption, there is a shift in the O–H stretching band from 3398 to 3391 cm^−1^ in the FTIR spectrum, which corresponds to the formation of O–H⋯N contacts. Two surface-sensitive techniques independently support that crystal violet is adsorbed onto the solid phase, not degraded or dissolved in solution. The XPS spectra of the dye-loaded adsorbent show an N 1s signal and lack the Cl 2p signal of the fresh material. SEM-EDX analysis of the same sample after adsorption showed the appearance of nitrogen (2.4 at%), as well as chlorine (1.2 at%), and a five-fold increase in the proportion of carbon, which is explained by the fact that crystal violet itself is mostly carbon, with N and Cl being counterions in the form of a chloride salt. The simultaneous presence of all these elements is further evidence that the dye–counterion pair was not altered but remained intact on the adsorbent surface. Another proof of this is desorption with a mild acid, resulting in the recovery of 89.4% ± 0.8% of the adsorbed dye; all the recovered samples showed identical spectrophotometer data to the original at 590 nm.

### 4.3 Vanadyl interaction (∼15%)

Based on our findings, it is suggested that the terminal vanadyl oxygen plays a role in the interaction mechanism. However, this phenomenon has not been previously observed for any other waste-derived adsorbents tested for CV sorption. After adsorption, there is a very modest 6 cm^−1^ red shift of the V=O stretching frequency from 459 to 453 cm^−1^, which points towards the surface interaction of this group. On the other hand, XPS data provide a limited explanation for the molecular-level interactions between the CV tertiary amino group and the Lewis acid and base sites of the vanadyl group through electron density donation. If these kinds of interactions were happening, a clear V 2p binding energy shift in the adsorbed material should have been the result; however, after checking, we found that the V 2p envelope was nearly the same in the material without and with CV. Thus, the most likely explanation is that it is a weak, non-covalent contact involving vanadyl oxygen, dipolar or hydrogen-bonding, which explains why it has not been interpreted as a coordination bond. It is the same type of physical, low-energy interaction that was supported by independent results of adsorption studies through the Dubinin–Radushkevich method, indicating *E* (mean free energy) = 3.10 kJ mol^−1^, which is a low energy for a physical interaction. This value is a far cry from the coordination energy of 8 kJ mol^−1^. In addition, this is a case of physical adsorption, since it has very low Δ*G*° values and is highly reversible. All pathways for this kind of sorption predict a very small vibrational shift with difference of ∼15% after accounting for the electrostatic and hydrogen-bonding contributions, makes it the most uncertain pathway among the three.

### 4.4 Preservation of the adsorbent framework

Raman spectroscopy reveals that the typical bands of the monoclinic vanadium oxide phase remain after adsorption, without any indication of phase transition, and the XRD analysis after adsorption indicates that the VO_2_ reflections have not changed. Combining these findings with the fact that the V 2p envelope was not altered, we can confidently conclude that the vanadium oxide framework is neither consumed nor does it undergo any chemical change during adsorption, and that adsorption only takes place on the surface. This is an essential feature that supports the excellent regeneration performance presented in Section 3.9; *i.e.*, that after fifteen consecutive cycles the material still retains 74.8% ± 1.1% of its initial capacity.

### 4.5 Mass transfer

Webber-Morris and Boyd's analysis of kinetic experiments done to full equilibrium recognized that kinetic curves can be described by three steps: (1) film diffusion (0–30 min) in which ∼60% of adsorption occurs through a sharp transport driven by external concentration gradients; (2) mesopore diffusion, which lasts from 30–120 min (∼30%) as a result of the penetration of dyes into 14.7 nm pores; and (3) micropore equilibration (120–240 min) as a process where the last accessible sites are filled. Under Box–Behnken design conditions, at a contact time of only half an hour, the film-diffusion stage alone gives the amount of adsorbed dye that was recorded by others. This is explained by the fact that for a mainly mesopore structure (mesopores constituting 81% of all pores), the mean pore diameter of 14.7 nm against the dimension of a CV molecule (∼1.6 nm) does not prevent diffusion, and intraparticle diffusion is not a limitation. This means that one cannot identify the pore network as a source of adsorption sites or an intrinsic source of capacity, since the former is merely a path to the latter. This is supported by a good correlation between the total rate of adsorption and that predicted by the pseudo-first-order model (*R*^2^ = 0.987, *k*_1_ = 0.0418 min^−1^, 0.3% error in *q*_e_), which suggests that the rate-limiting step is adsorption onto the surface of the active sites rather than bond making.

### 4.6 Reversibility and regeneration

The minimal changes in Δ*G*° that indicate physisorption in the low-energy range allow for the physical adsorption of molecules and the regeneration of the system. The regeneration process is supported by the thermodynamic reversibility that comes from the low magnitude of Δ*G*°. For regeneration, the acid washing protonates the surface, which cancels the electrostatic charge contribution; heating up to 300 °C removes any remaining organics, and the surface hydroxylation is recovered in the cooling phase. These methods in combination give 89.4% ± 0.8% of adsorption in the first cycle and 74.8% ± 1.1% retention of the initial capacity after fifteen cycles, which is a significant improvement and much better than the five-cycle benchmark usually considered the threshold for economic viability.

### 4.7 Summary of the mechanistic account

An overview of the mechanistic explanation is as follows. The evidence points to a physisorption mechanism that is dominated by surface chemistry rather than porosity. The main driving force in this mechanism is electrostatic attraction between a negatively charged surface and the cationic dye, which is reinforced by hydrogen bonding to surface hydroxyls and a weaker interaction involving the vanadyl oxygen. The vanadyl contribution is supported by a single vibrational shift and is indicated by XPS to be a non-charge-transfer in character, while the electrostatic and hydrogen-bonding contributions are confirmed by direct and independent measurements. The quantitative allocation of the three pathways should be considered indicative rather than precise, and it is a logical extension of this work to refine it, for instance by monitoring the surface V^4+^/V^5+^ distribution over subsequent regeneration cycles.

## 5. Comparative performance

The comparative analysis presented in Table 18 is clearly separated into four benchmarking categories: (A) spent industrial catalyst adsorbents, which are most directly comparable to SVC in terms of material origin, surface chemistry, and absence-of-pretreatment value proposition; (B) adsorbents derived from metallurgical and industrial waste, which frame the industrial waste valorization context; (C) adsorbents derived from agricultural and bio-waste, which represent the wider landscape of low-cost alternatives; and (D) engineered and nanomaterial-based adsorbents, which represent the upper performance boundary. The benchmarking parameters that are most pertinent to real-world implementation choices include *q*_max_, BET surface area, pretreatment needs, operating conditions, CV removal efficiency, and regeneration performance.

**Table 18:** Comparison of adsorbent performance for dye removal from aqueous solutions^a^

Adsorbent	Pretreatment burden	Dose (g L^−1^)	*q*_max_ (mg g^−1^)	*q*_e_ at *C*_0_ ≈ 40 mg L^−1^	Contact time	Removal (%)	Cost tier	Ref.
**Group I—zero pretreatment (used as-collected industrial waste)**								
Spent FCC catalyst	None	NR	69.4 (L)	NR	NR	High (% NR)	✦ free	61
SVC**—**spent V_2_O_5_ contact-process catalyst (this study)	None	6.3	21.8 (L, NL)	6.1	0.5 h	94.5 ± 0.3	✦ free	This work
DCI foundry dust (MgO-rich)	None	6.2	116.85 (D–R)	6.5	0.5 h	93.7	✦ free	62

**Group II—single-step mild pretreatment**								
Surface-enhanced coal fly ash	Mild	1.25	NR	NR	1 min	∼97.5 (*C*_0_ = 10)	✦✦ Low	63
Acid-activated red mud (ASRM)	Mild	NR	60.5 (L)	NR	NR	High (% NR)	✦✦ Low	64
Sugarcane bagasse biosorbent	Mild	NR	61.35 (L)	NR	40 min	High (% NR)	✦✦ Low	65
Acorn waste (BBD-optimised)	Mild	NR	NR	NR	NR	>90	✦✦ Low	66

**Group III—two-step moderate pretreatment**								
Fly ash–steel slag geopolymer	Moderate	NR	6.11 (L)	NR	NR	91.66	✦✦✦ Med	67
Pomegranate peel activated carbon	Moderate	NR	35.71 (L, 25 °C); 90.91 (40 °C)	NR	60–90 min	High (% NR)	✦✦✦ Med	68
Banana stem biochar (BSB350)	Moderate	0.025	∼208.33 (L)	NR	25–60 min	∼95–96	✦✦✦ Med	69

**Group IV—multi-step synthesis or calcination ≥500 °C**								
MgO nanorods (coprecipitation)	Intensive	0.4	493.90 (L, BF)	NR	236 min	Selective	✦✦✦✦ High	70
Magnetic Fe_3_O_4_ nanoparticles	Intensive	0.3	114.8 (L)	NR	120 min	High (% NR)	✦✦✦✦ High	71
Sulfonated graphene oxide	Intensive	Mg-scale	97.7 (298 K); 202.5 (308 K)	NR	NR	Very high	✦✦✦✦✦ V. high	72
Neoteric magnetic nanostructure	Intensive	3.33	∼19.45 (L)	NR	180 min	∼92–93	✦✦✦✦ High	73
FCOB biosorbent (curcumin/PAA)	Intensive	NR	≥275	NR	NR	High (% NR)	✦✦✦✦ High	74

a NR = not reported in the cited source. NQ = not quantified. L = Langmuir; NL = nonlinear regression; D–R = Dubinin–Radushkevich; BF = basic fuchsine (structurally analogous cationic triphenylmethane dye). Pretreatment burden: none = used as collected with no modification; mild = single washing, drying, or mild chemical step; moderate = two sequential steps (*e.g.* pyrolysis + activation); intensive = multi-step chemical synthesis or calcination ≥500 °C. Cost tier reflects adsorbent preparation cost relative to SVC: ✦ free; ✦✦ low (<$100 per MT); ✦✦✦ medium ($100–500 per MT); ✦✦✦✦ high (>$500 per MT); ✦✦✦✦✦ very high (>$2000 per MT).

Two major points come to light from the comparison. (i) Within Category A, SVC appears to be the first spent contact-process vanadium catalyst adsorbent for CV removal. It should be noted that spent FCC catalyst (Category A, entry 1) has been shown to have an even higher Langmuir *q*_max_ (69.4 mg g^−1^) with CV; however, the material has a much higher BET surface area (typically >100 m^2^ g^−1^) and no vanadyl coordination sites; SVC provides a mechanistic pathway, V^4+^=O coordination with CV nitrogen, that has not been reported for other waste-derived adsorbents. (ii) Within Category B, all metallurgical waste entries need at least one pre-treatment step (alkali activation, acid activation, or thermal treatment), while only SVC and DCI waste are used directly without any modifications. SVC offers 94.5% removal in a 0.5 h contact time, the shortest equilibrium time in the whole set, because the dose–time interaction (BD) at the optimized dose of 6.3 g L^−1^ enables rapid film diffusion alone to bring near-complete uptake within 30 minutes.

The theoretical capacity obtained from the Dubinin–Radushkevich isotherm (8.4 mg g^−1^ from *D*, *R*) and the Langmuir nonlinear fit (21.8 mg g^−1^) are based on different physical assumptions and, hence, they should not be directly compared among themselves or with Langmuir capacities from other studies without proper consideration. The Freundlich isotherm, which gave the best empirical agreement with the experimental data (*R*^2^ = 0.984, RMSE = 0.174, and *χ*^2^ = 0.055), does not result in a single *q*_max_ value; it does not define one at all. The measured equilibrium capacity at a representative CV concentration of 40 mg L^−1^ in optimized conditions (dose = 6.3 g L^−1^, 1, pH 8.0, 25 °C, 0.5 h) is *q*_e_ ≈ 6.1 ± 0.2 mg g^−1^ (*n* = 3), which is the most practically significant reference for comparison with the Langmuir-based *q*_max_ values that are reported at similar operating concentrations. SVC has the distinct feature of zero preparation cost plus the presence of vanadyl coordination sites that are not found in any other entries in the table.

The fact that SVC is not a high-capacity adsorbent must be made very clear. In comparison to chemically activated biochars (∼208 mg g^−1^ at 0.025 g L^−1^), engineered nanomaterials (97–275 mg g^−1^ at 0.3–3.3 g L^−1^), and synthesized MgO (494 mg g^−1^ at 0.4 g L^−1^), the working capacity (*q*_e_ = 6.1 ± 0.2 mg g^−1^ at *C*_0_ = 40 mg L^−1^) and the Langmuir monolayer capacity from nonlinear regression (21.8 mg g^−1^) should not be interpreted as proof of high intrinsic affinity because the 94.5% removal reported here is attained at a relatively high adsorbent dose (6.3 g L^−1^) and a moderate initial concentration (38.5 mg L^−1^). Banana stem biochar achieves about ten times the capacity of SVC at about 1/205 of the dose. Therefore, capacity weighed against preparation cost and pretreatment burden rather than capacity alone is the relevant comparison. Pyrolysis, calcination above 500 °C, or multi-step chemical synthesis are required for all materials in the upper capacity tier, and a high dose that is prohibitively expensive for activated carbon at $2200 per ton is acceptable for a material that requires no preparation and has no purchase cost. SVC (6.1 ± 0.2 mg g^−1^ (*n* = 3) at 6.3 g L^−1^) performs similarly to MgO-rich foundry dust (6.5 mg g^−1^ at 6.2 g L^−1^) within the subset of adsorbents deployed as-collected without modification, and this is the proper benchmark class for the material. Notably, fly ash–steel slag geopolymer, which needs CO_2_ curing and alkali activation, achieves 6.11 mg g^−1^, which is nearly the same as SVC without pretreatment.

## 6. Conclusions

Vanadium pentoxide, a catalyst left over from the sulfuric acid contact process, is usually buried in landfills, amounting to about 150 000 to 200 000 tons per year. The use of vanadium pentoxide as a free, chemically unprocessed, efficient, spent vanadium pentoxide catalyst-based (SVC) adsorbent for crystal violet (CV) removal, which, in the case of materials derived from other wastes, has not been found in this mechanistic mode, is demonstrated here. XRD analysis detected monoclinic VO_2_ as the major crystalline form of vanadium. XPS showed that vanadium on the surface is mainly V^4+^, and this partial reduction probably happened because the original V_2_O_5_ was reduced in the SO_2_ → SO_3_ catalytic cycle. Raman spectroscopy confirmed and further proved that there is no transformation on adsorption of the crystal phases and that the vanadyl complexes of this reduced form of the catalysts are the ones responsible for the adsorption process through a weak, non-charge-transfer type of interaction (as the vanadium 2p core level XPS envelope remains mostly unchanged post-adsorption). Textural data obtained by BET surface area (mesopores 81%, surface 11.4 ± 0.3 m^2^ g^−1^, average pore size 14.7 nm), and a measured zeta potential of −10.58 mV at pH 7; pH_PZC_ ≈ 5.2 (*i.e.* the material surface is negatively charged in a pH range of 6–9) indicate that textural features (and hence adsorbent site availability) and the sign of charges between the material and the adsorbate are sufficient. Moreover, this is accomplished at the low surface area of just 11.4 m^2^ g^−1^, corresponding to around 0.54 mg m^−2^, *i.e.*, about one twentieth that of the areal loading of a standard benchmark material, being an MgO-rich foundry dust. Therefore. the adsorption properties of such a low surface area material are more determined, or at least mainly a function of material chemistry rather than of material porosity. A Box–Behnken design (*R*^2^ = 0.9926) revealed that the adsorbent dose, time interaction (*F* = 142.3, *p* < 0.0003, 23.9% of model SS) is the main process variable, and the optimized conditions (*C*_0_ = 38.5 mg L^−1^, dose = 6.3 g L^−1^, 150 rpm, 0.5 h) showed 94.5% ± 0.3% removal (*n* = 3) experimentally. Nonlinear regression identified the Freundlich model as the best isotherm fit (*R*^2^ = 0.984, *χ*^2^ = 0.055, 1/*n* = 0.578) and pseudo-first-order kinetics as rate-governing (*R*^2^ = 0.987, *k*_1_ = 0.0418 min^−1^, 0.3% *q*_e_ error), both consistent with heterogeneous physisorption on a multi-site surface. Thermodynamic parameters (Δ*H*° = +18.6 kJ mol^−1^; Δ*S*° = +67.5 J mol^−1^ K^−1^; Δ*G*° = −0.84 to −2.87 kJ mol^−1^) confirm endothermic, entropy-driven spontaneous adsorption across 288–318 K, with the small Δ*G*° magnitude explaining the 89.4% ± 0.8% (*n* = 3) first-cycle desorption efficiency achieved under mild acid-thermal regeneration. Three parallel surface interaction pathways operate simultaneously: electrostatic attraction (∼60%, confirmed by ionic-strength suppression), hydrogen bonding (∼25%, indicated by the FTIR O–H shift), and a proposed vanadyl coordination contribution (∼15%, indicated by the FTIR V=O shift). Retention of intact dye on the surface is established by EDX detection of nitrogen and chlorine and by quantitative recovery of the dye on acid desorption. ICP-OES leaching confirmed vanadium release of 12 µg L^−1^ at working conditions (pH 8, 25 °C, 4 h), which is below the California OEHHA 15 µg L^−1^ benchmark but above the German guidance value (4 µg L^−1^), so operation must be restricted to pH 6–9, and adsorbent replacement at cycle 12 is recommended to maintain both capacity (∼79%) and leaching safety. Combined acid-thermal regeneration still works effectively after 15 consecutive cycles, keeping up to 74.8% ± 1.1% (*n* = 3) adsorption capacity (∼1.68% loss per cycle), with characterization of post-cycle XRD and BET identifying K_2_SO_4_ crystallite formation and surface area loss (11.4 → 7.8 m^2^ g^−1^) as the main deactivation paths. At 2026 global commodity prices, the estimated unit treatment cost of the base case is $1.53 m^−3^, accounting for a 60–80% reduction in cost with respect to the commercial activated carbon used in middle-income country settings. Altogether, these findings put forward SVC as a circular economy material with a scientifically distinct mechanism, well-delineated safety boundaries, and proven economic feasibility at the proof-of-concept stage. The fixed-bed column work and the quantitative vanadyl site characterization through the regeneration cycles are the most important next steps in commercial validation.

## Conflicts of interest

The authors declare no competing financial interests.

## Data Availability

The data supporting the findings of this study are available within the article. Additional raw data, including experimental datasets, characterization resultsare available from the corresponding author upon reasonable request.

## References

[cit1] Kolya H., Kang C. W. (2024). Toxics.

[cit2] Kusumlata B. A., Kumar A., Gautam S. (2024). Limnol. Rev..

[cit3] Sifat M., Shin E., Schevon A., Ramos H., Pophali A., Jung H. J., Halada G., Meng Y., Olynik N., Sprouster D. J., Kim T. (2024). Catalysts.

[cit4] Aijaz S. A., Shafi Z., Shahid M. (2026). RSC Adv..

[cit5] Liu Y., Chen J., Duan D., Zhang Z., Liu C., Cai W., Zhao Z. (2024). Sustainability.

[cit6] Dutta S., Adhikary S., Bhattacharya S., Roy D., Chatterjee S., Chakraborty A., Banerjee D., Ganguly A., Nanda S., Rajak P. (2024). J. Environ. Manage..

[cit7] qiong Gao Y., xuan Li K., Zeng Q., xiang Tian F., ning Fan Z., yi Xing G., yun Gao N. (2024). J. Water Process Eng..

[cit8] Lee D. J., Kim J. Y., Park J., Bin Choi Y., Kim J. K., Choi H., Tsang Y. F., Kwon E. E. (2024). Chemosphere.

[cit9] Sanap S., Pote D., Sorate D., U B., Ingole D., Tavashi S. (2025). Int. J. Environ. Sci..

[cit10] Zhang W., Ji Y., Zhang J., Zhang H., Chang C., Wang Z. (2024). J. Environ. Chem. Eng..

[cit11] Ranjan P., Saptal V. B., Bera J. K. (2022). ChemSusChem.

[cit12] Sun S., Zhuang P., Li Z., Mo H. (2024). Environ. Technol. Innov..

[cit13] Bux C., Zizzo G., Roe B. E., Amicarelli V. (2025). J. Clean. Prod..

[cit14] Sofyana A., Muslim A., Supardan M. D., Ambarita A. C., Arahman N. (2024). Case Stud. Chem. Environ. Eng..

[cit15] Barua S. (2025). Int. J. Technol. Manag. Hum..

[cit16] Wen S., Cheng W. C., Li D., Hu W. (2023). Environ. Technol. Innov..

[cit17] Li R., Zhang L., Zhang Y., Zhang Z., Guan Y. (2024). Chem. Eng. J..

[cit18] Wu X., Cai J., Yang J., Ma Y., Li M., Wu F. (2025). J. Clean. Prod..

[cit19] Long Y., Song Q., Huang B., Zeng X., Wu H. (2024). Resour. Conserv. Recycl..

[cit20] Shahbazi M., Jäger H., Ettelaie R., Chen J., Kashi P. A., Mohammadi A. (2024). Adv. Colloid Interface Sci..

[cit21] Zhao J., Zhang X., Yang F., Ai Y., Chen Y., Pan D. (2024). ACS Omega.

[cit22] ASTM D7928-17 , ASTM International, 2017, preprint, DOI: 10.1520/D7928-17

[cit23] Karan K. K., Masto R. E., Agarwalla H., Bari S., Kumar M., Gopinathan P., Hazra B., Saha S., Maity S. (2023). Environ. Sci.:Adv..

[cit24] Schroeder P. A. (1993). Clays Clay Miner..

[cit25] Thommes M., Kaneko K., Neimark A. V., Olivier J. P., Rodriguez-Reinoso F., Rouquerol J., Sing K. S. W. (2015). Pure Appl. Chem..

[cit26] Osterrieth J. W. M., Rampersad J., Madden D., Rampal N., Skoric L., Connolly B., Allendorf M. D., Stavila V., Snider J. L., Ameloot R., Marreiros J., Ania C., Azevedo D., Vilarrasa-Garcia E., Santos B. F., Bu X. H., Chang Z., Bunzen H., Champness N. R., Griffin S. L., Chen B., Lin R. B., Coasne B., Cohen S., Moreton J. C., Colon Y. J., Chen L., Clowes R., Coudert F. X., Cui Y., Hou B., D'Alessandro D. M., Doheny P. W., Dinca M., Sun C., Doonan C., Huxley M. T., Evans J. D., Falcaro P., Ricco R., Farha O., Idrees K. B., Islamoglu T., Feng P., Yang H., Forgan R. S., Bara D., Furukawa S., Sanchez E., Gascon J., Telalovic S., Ghosh S. K., Mukherjee S., Hill M. R., Sadiq M. M., Horcajada P., Salcedo-Abraira P., Kaneko K., Kukobat R., Kenvin J., Keskin S., Kitagawa S., Otake K. I., Lively R. P., DeWitt S. J. A., Llewellyn P., V Lotsch B., Emmerling S. T., Putz A. M., Marti-Gastaldo C., Padial N. M., Garcia-Martinez J., Linares N., Maspoch D., Suarez Del Pino J. A., Moghadam P., Oktavian R., Morris R. E., Wheatley P. S., Navarro J., Petit C., Danaci D., Rosseinsky M. J., Katsoulidis A. P., Schroder M., Han X., Yang S., Serre C., Mouchaham G., Sholl D. S., Thyagarajan R., Siderius D., Snurr R. Q., Goncalves R. B., Telfer S., Lee S. J., Ting V. P., Rowlandson J. L., Uemura T., Iiyuka T., van der Veen M. A., Rega D., Van Speybroeck V., Rogge S. M. J., Lamaire A., Walton K. S., Bingel L. W., Wuttke S., Andreo J., Yaghi O., Zhang B., Yavuz C. T., Nguyen T. S., Zamora F., Montoro C., Zhou H., Kirchon A., Fairen-Jimenez D. (2022). Adv. Mater..

[cit27] Particle size analysis-Dynamic light scattering (DLS) COPYRIGHT PROTECTED DOCUMENT, 2017

[cit28] Colloidal systems-Methods for zeta-potential determination-COPYRIGHT PROTECTED DOCUMENT, 2012

[cit29] Veerasingam S. , Ranjani M., Venkatachalapathy R. and Vethamony P., Groundwater Contamination in Coastal Aquifers: Assessment and Management, 2022, 135–139

[cit30] Cartwright H. (2006). Chem. Educ..

[cit31] Vyazovkin S., Burnham A. K., Criado J. M., Pérez-Maqueda L. A., Popescu C., Sbirrazzuoli N. (2011). Thermochim. Acta.

[cit32] Shvets P., Krylov A., Maksimova K., Goikhman A. J. (2024). J. Raman Spectrosc..

[cit33] Mussi V., Bovino F. A., Falsini R., Daloiso D., V Lupo F., Kunjumon R., Voti R. L., Cesca T., Macaluso R., Sibilia C., Mattei G. (2024). Sci. Rep..

[cit34] Biesinger M. C. (2017). Surf. Interface Anal..

[cit35] Biesinger M. C. (2022). Appl. Surf. Sci..

[cit36] Revellame E. D., Fortela D. L., Sharp W., Hernandez R., Zappi M. E. (2020). Clean. Eng. Technol..

[cit37] Wang Y., Wang C., Huang X., Zhang Q., Wang T., Guo X. (2024). Chemosphere.

[cit38] Danat B. T., Wuana R. A., Chahul H. F., Iorungwa M. S. (2026). Appl. Water Sci..

[cit39] Wu C.-W., V Kothare M., Sircar S. (2014). Ind. Eng. Chem. Res..

[cit40] Liu Y., Chen J., Duan D., Zhang Z., Liu C., Cai W., Zhao Z. (2024). Sustainability.

[cit41] Denton J. E. , Hickox W. H., Spath D. P., V Alexeeff G., Fan A. M. and Howd R. A., Office of Environmental Health Haza. Assessment, 2000

[cit42] German Environmental Agency , Vanadium, World Health Organization, 1989

[cit43] Guidelines for Drinking-Water Quality FIRST ADDENDUM TO THIRD EDITION Volume 1 Recommendations WHO Library Cataloguing-in-Publication Data, 2006

[cit44] EPA , Environmental Monitoring Systems Laboratory, 1993, 1–15

[cit45] Egyptian Environmental Agency , LAW NUMBER 4 of 1994* PROMULGATING the ENVIRONMENT LAW and its EXECUTIVE REGULATION EGYPT * in Case of Difference of Interpretation, the Arabic Text Will Prevail, Egypt, 1994

[cit46] Jevremović A., Ranković M., Janošević Ležajić A., Uskoković-Marković S., Nedić Vasiljević B., Gavrilov N., Bajuk-Bogdanović D., Milojević-Rakić M. (2025). Sustainable Chem..

[cit47] Zhao J., Yang X., Yang Y., Liu L., Lin Y., Xie L., Chai X., Xu K., Du G., Zhang L. (2024). Chem. Eng. J..

[cit48] Al Amayreh H. H., Khalaf A., Hawwari M. I., Hourani M. K., Al Bawab A. (2023). Materials.

[cit49] Wang Y., Wang C., Huang X., Zhang Q., Wang T., Guo X. (2024). Chemosphere.

[cit50] Vitek R., Masini J. C. (2023). Heliyon.

[cit51] Chu K. H., Hashim M. A., Zawawi M. H., Bollinger J. C. (2025). J. Environ. Chem. Eng..

[cit52] Chu K. H., Hashim M. A., Hayder G. (2024). J. Mol. Liq..

[cit53] Hagiwara T., Motomizu S. (1994). Bull. Chem. Soc. Jpn..

[cit54] Schäfer A., Giannini S., Strelnikov D., Mohr T., Santoro F., Cerezo J., Kappes M. M. (2024). Phys. Chem. Chem. Phys..

[cit55] U. Epa and O. of Resource Conservation , RCRA Resource Conservation and Recovery Act Orientation Manual 2014 RCRA Orientation Manual 2014 Reducing Priority Chemicals Saving Resources and Energy Managing Materials Safely Forming Partnerships Promoting Recycling and eCycling Recycled/Recyclable-Printed with Vegetable Oil Based Inks on 100% (Minimum 50% Postconsumer) Recycled Paper, 2014

[cit56] Johari A. , Kumar J., Gautam P. and Ranjan V., Hydrochloric Acid Price Trend 2026 — Forecast and Analysis, Expert Market Research, 2026

[cit57] IMARC Group, Hydrochloric Acid Price Index, Trend and Forecast, 2026

[cit58] IMARC Group, Activated Carbon Prices, Chart, Index and Forecast 2026

[cit59] International Energy Agency, Electricity 2026, Paris, 2026

[cit60] GlobalPetrolPrices.com, World Electricity Prices — Global Business Average

[cit61] Gameiro T., Costa C., Labrincha J., Novais R. M. (2023). Mater. Today Sustain..

[cit62] Ibrahim I. M., Turky A. M., Mostafa N. Y., Roushdy M. H. (2025). RSC Adv..

[cit63] Dwivedi D. K., Sharma B., Bochare R., Dwivedi M. K. (2025). Total Environ. Eng..

[cit64] Tan D., Yuan P., Annabi-Bergaya F., Liu D., He H. (2014). Appl. Clay Sci..

[cit65] Imame S. , Loukili K., Atlas H., Legsaier M., El Bakkali K. M., Sadoq M. and Boukhlifi F., in E3S Web of Conferences, EDP Sciences, 2025, vol. 649

[cit66] Loukili K. , Imame S., El Bakkali M. K., Lagseir M., Sadoq M., Atlas H., El Bourachdi S., Lahkimi A. and Boukhlifi F., in E3S Web of Conferences, EDP Sciences, 2025, vol. 649

[cit67] Hossain M. D., Ling X., Sun S., Wadjou C., Ahmed M., Tanvir S. A., Zhang L. (2025). J. Environ. Earth Sci..

[cit68] Mehta D., Dave P. N., Kumar V. V. (2025). RSC Adv..

[cit69] Baharim N. H., Sjahrir F., Mohd Taib R., Idris N., Tuan Daud T. A. (2023). Chem. Eng. Trans..

[cit70] Ghoniem M. G., Ali F. A. M., Abdulkhair B. Y., Elamin M. R. A., Alqahtani A. M., Rahali S., Ben Aissa M. A. (2022). Nanomaterials.

[cit71] Hamidzadeh S., Torabbeigi M., Shahtaheri S. J. (2015). J. Environ. Health Sci. Eng..

[cit72] Oluwasina O. O., Adelodun A. A., Oluwasina O. O., Duarte H. A., Olusegun S. J. (2024). Sci. Rep..

[cit73] Avugadda S. K., Wickramasinghe S., Niculaes D., Ju M., Lak A., Silvestri N., Nitti S., Roy I., Samia A. C. S., Pellegrino T. (2021). Nanomaterials.

[cit74] Mosaffa E., Patel R. I., Banerjee A., Basak B. B., Oroujzadeh M. (2024). RSC Adv..

